# Correlated miR-mRNA Expression Signatures of Mouse Hematopoietic Stem and Progenitor Cell Subsets Predict “Stemness” and “Myeloid” Interaction Networks

**DOI:** 10.1371/journal.pone.0094852

**Published:** 2014-04-18

**Authors:** Diane Heiser, Yee Sun Tan, Ian Kaplan, Brian Godsey, Sebastien Morisot, Wen-Chih Cheng, Donald Small, Curt I. Civin

**Affiliations:** 1 Center for Stem Cell Biology and Regenerative Medicine, Departments of Pediatrics and Physiology, University of Maryland School of Medicine, Baltimore, Maryland, United States of America; 2 Department of Oncology, Sidney Kimmel Comprehensive Cancer Center, Johns Hopkins University School of Medicine, Baltimore, Maryland, United States of America; Rutgers-Robert wood Johnson Medical School, United States of America

## Abstract

Several individual miRNAs (miRs) have been implicated as potent regulators of important processes during normal and malignant hematopoiesis. In addition, many miRs have been shown to fine-tune intricate molecular networks, in concert with other regulatory elements. In order to study hematopoietic networks as a whole, we first created a map of global miR expression during early murine hematopoiesis. Next, we determined the copy number per cell for each miR in each of the examined stem and progenitor cell types. As data is emerging indicating that miRs function robustly mainly when they are expressed above a certain threshold (∼100 copies per cell), our database provides a resource for determining which miRs are expressed at a potentially functional level in each cell type. Finally, we combine our miR expression map with matched mRNA expression data and external prediction algorithms, using a Bayesian modeling approach to create a global landscape of predicted miR-mRNA interactions within each of these hematopoietic stem and progenitor cell subsets. This approach implicates several interaction networks comprising a “stemness” signature in the most primitive hematopoietic stem cell (HSC) populations, as well as “myeloid” patterns associated with two branches of myeloid development.

## Introduction

microRNAs (miRs) have emerged as novel regulators in many physiologic and pathophysiologic processes, and studies of the accessible and tractable hematopoietic system have identified many individual miRs exerting control over proliferation and differentiation. Acting to repress translation or lead to degradation of target mRNAs through partially complementary binding, these 18–24 base pair molecules exert a post-transcriptional layer of control over differentiation in several hematopoietic lineages.

In myelopoiesis, miR-223 has been shown to regulate granulocyte development in both humans and mice [1], [2], while the clustered miRs 144 and 451 are important regulators of erythropoiesis [3]. miRs also play important roles in lymphoid differentiation, with miR-155 regulating T helper cell differentiation and germinal center responses [4], miR-150 regulating Natural Killer (NK) and invariant NK T cells [5], and the miR-17∼92 cluster being essential for B cell development [6]. Less is known about miR control over hematopoietic stem cell maintenance and self-renewal. Conditional Dicer knockout mice using either mx1-CRE or the HSC-specific vav-CRE have demonstrated that HSCs are dependent on this miR-processing enzyme, indicating that one or more miRs are necessary for hematopoiesis [7], [8]. While miR-125a has been shown to regulate the size of the HSC pool in mice, it remains unknown which miRs are necessary for HSC maintenance and self-renewal [7].

While these studies of individual miRs have revealed much about control of hematopoietic development, there have been no comprehensive studies of miR *networks* that operate during the early stages of hematopoietic differentiation and maturation. Here we create a map of global miR expression in each stage of early hematopoietic stem and progenitor cell development, with a focus on the myeloid branch of differentiation. We have profiled miR expression in 6 Hematopoietic Stem/Progenitor Cell (HSPC) populations: Long-Term Hematopoietic Stem Cell (LT-HSC), Short-Term HSC (ST-HSC), Multipotent Progenitor (MPP), Common Myeloid Progenitor (CMP), Granulocyte-Monocyte Progenitor (GMP) and Megakaryocyte-Erythroid Progenitor (MEP) [9]. We then correlated microarray values with qRT-PCR-measured absolute copy number per cell to generate a database of estimated miR expression in each cell type. As data is emerging in the literature that a given miR must be expressed above a certain intracellular threshold level to exert a substantial functional effect, this absolute quantification database provides a valuable resource for the identification of miRs with functional roles in these rare stem and progenitor populations [10], [11], [12].

Further, we have combined this miR expression data with mRNA expression data from the same populations to create a global miR-mRNA interaction database. By using a novel Bayesian approach which takes into account the ordered nature of hematopoietic differentiation, we created an algorithm to identify inverse expression correlations between miR-mRNA pairs [13]. In combination with two existing target prediction algorithms (TargetScan and MiRanda), this program was used to identify a global network of interactions between miRs and mRNAs during early hematopoietic differentiation.

## Results

### Isolation of early hematopoietic stem and progenitor populations

**Figure 1:**
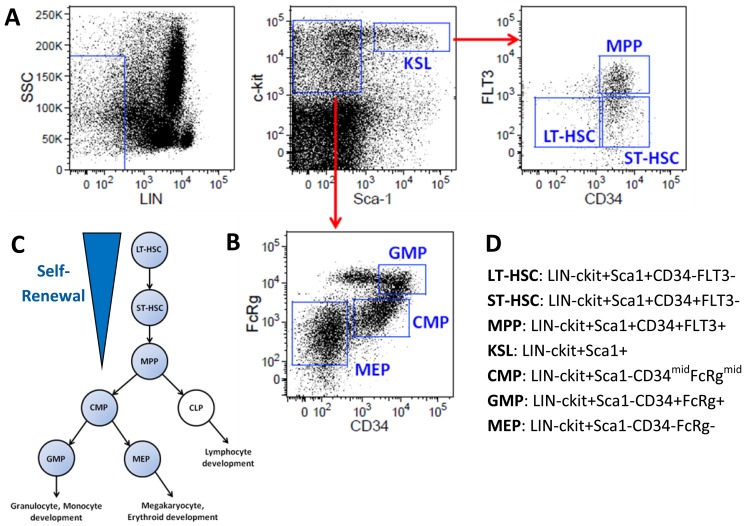
Isolation of mouse hematopoietic stem and progenitor subsets from whole bone marrow. Gating strategy used to isolate HSPC populations in A) Hematopoietic Stem Cell sort and B) Myeloid Progenitor sort. C) Hierarchy used in subsequent informatics analysis D) Summary of defining cell surface markers used to separate HSPC subsets.

From among the several methods utilized to isolate hematopoietic stem cells (HSCs) on the basis of differential cell surface antigen expression, we chose a strategy capable of separation of both HSCs and multiple defined progenitor subtypes [14], [15]. Mouse bone marrow was first depleted of mature cells expressing “lineage” antigens, followed by immunomagnetic enrichment of cells expressing c-kit, to obtain a bulk population enriched in Hematopoietic Stem and Progenitor Cells (HSPCs) (Figure 1A). Populations enriched in hematopoietic stem cells (“HSC subsets”: Long-Term HSCs, Short-Term HSCs and Multipotent Progenitors) were isolated from bulk HSPCs in a single FACS sort according to the schematic shown in Figure 1B. Myeloid progenitor populations (Common Myeloid Progenitors, Granulocyte-Monocyte Progenitors and Megakaryocyte-Erythroid Progenitors) were isolated in a separate FACS sort from bulk HSPCs along with the more heterogeneous KSL population (c-Kit+, Sca-1+, Lineage−), as shown in Figure 1C. The surface marker separation strategy used for each individual population is summarized in Figure 1D, and has been previously described [14], [15].

RNA was isolated from each indicated FACS-sorted HSPC subset and profiled for miR and mRNA expression using Agilent single-color miR arrays and low input 4×44k mRNA arrays.

### Quantification of miR copy number per cell in HSPC subsets

**Figure 2:**
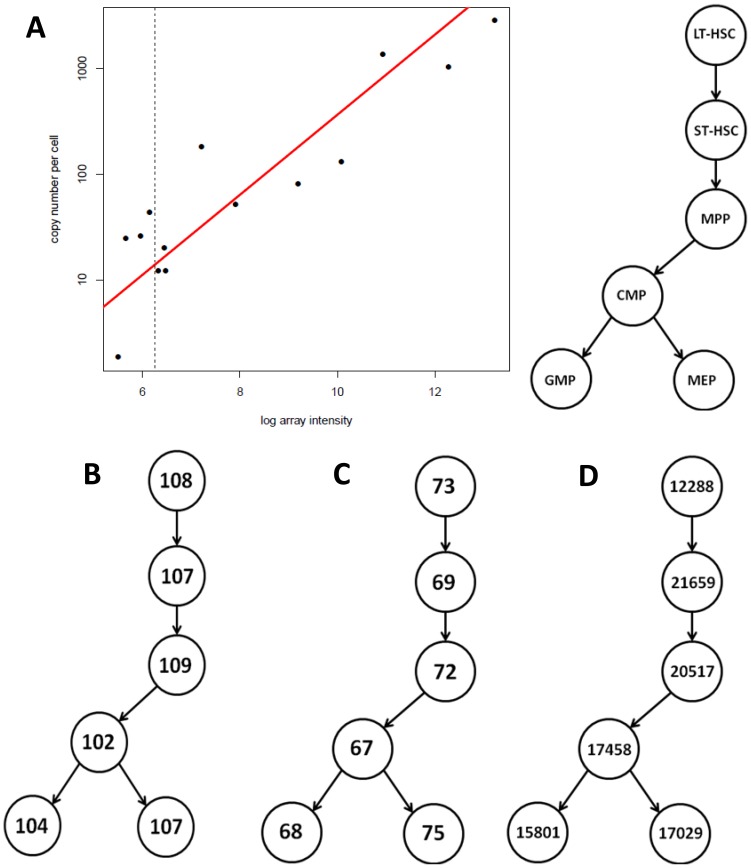
Quantification of miR copy number is used to determine functional levels of expression in HSPC subsets. a) copy number per cell was determined in the GMP subset for 15 representative miRs, and plotted against normalized array value. Regression analysis was used to infer the relationship between the two values (best-fit line appears in red), and from this we estimated the array value above which we are 95% certain that a miR with >100 copies per cell would appear. That is, we estimated a one-sided 95% confidence interval of array values for miRs with 100 copies per cell or greater. Thus, miRs with >100 copies per cell have array values over the estimated cut-off (6.25) with significance level p<0.05. b) number of individual miRs c) number of miR families represented d) number of mRNAs expressed in each HSPC population, as determined by having at least one probe significantly higher than “dark” control probes (p<0.01), as determined by an unpaired t-test comparing replicates of a unique probe's values to all dark control probes.

As emerging data in the literature suggests that miRs must be present at levels above a certain threshold in order to functionally repress their targets [10], [11], [12], we sought to create a resource describing the absolute copy number per cell of each individual miR in each HSPC subset. To this end, microarray expression values were subjected to quantile normalization using Limma software, and normalized values were correlated with copy number per cell as determined by Taqman qPCR [16]. Standard curves were constructed using known concentrations of synthesized RNA oligos for 15 representative miRs (selected over a range of array expression values), and then used to calculate miR copy number per cell. We used the GMP subset for this validation process, as this was a relatively abundant progenitor population. All HSPC populations measured yielded 5–8 pg of total RNA per cell. 8 pg per cell was chosen to calculate absolute copy number for all populations, to err on the side of inclusion of miRs expressed near the threshold. For 15 individual miRs with a range of array values, normalized array intensity was plotted against qPCR-measured absolute copy number per cell. In order to minimize confounding effects of GC content we chose a range of miRs from high to low GC contents. We then performed a regression analysis in order to set a copy number threshold based on normalized array values (Figure 2A). The threshold for miRs predicted to be expressed at functional levels was set at 100 copies per cell, based on recent reports [10], [11]; specifically, we set a rather inclusive threshold at 95% confidence to include all miRs expressed at ≥100 copies per cell (normalized array intensity value ≥6.25, based on the regression analysis) (Table S1). In this manner, we are able to say with 95% certainty that a miR present at >100 copies per cell will appear above our threshold (p<0.05) and will be included in subsequent analyses. While this precise threshold level of 100 miR molecules per cell is likely not the absolute functional threshold for every cell and every microRNA, applying this threshold substantially narrows our “miRs of interest” group for subsequent analyses. By including this information on absolute copy number, and therefore excluding those miRs that are likely to be expressed well below levels where they are likely to repress their targets substantially, we are able to focus our algorithm on the miRs most likely to be playing a functional role in HSC maintenance and early hematopoietic differentiation. In this manner, estimates of absolute copy number act as an additional factor in the computational global analysis of miR and mRNA expression data, potentially reducing false positives of miRs expressed below a functional level.

Interestingly, all HSPC subsets expressed a similar total number of individual miRs at supra-threshold levels, and the total number of miR families expressed at supra-threshold levels was also constant throughout early hematopoietic development (Figure 2B,C). This suggests that at least in the hematopoietic system, stem cells and early progenitors do not differ greatly in overall levels of microRNA present. Individual miRs as well as specific families expressed in each HSPC population (as well as normalized array intensities) are described in Table S1. 98 different miR families were found to be expressed at supra-threshold levels in at least one HSPC subset; 46 families were expressed at supra-threshold levels in all six stem and progenitor cell subsets; 14 families were expressed at supra-threshold levels in only one subset, 7 of which were unique to the MEP; 12 families were expressed at supra-threshold levels in all but one of the individual populations (7 of which were absent only in the MEP), and the remaining 26 were expressed at supra-threshold levels in some combination (but not all) of the HSPC populations. Differences in miR family expression between the HSPC populations are described in detail in Table S2. Since there is no consensus on the number of mRNA molecules necessary for function, the number of individual mRNAs expressed in each population was determined simply by the presence of at least one probe significantly higher than “dark” negative control probes (p<0.01), as determined by an unpaired t-test comparing replicates of a unique probe's values to all dark control probes (Figure 2D).

### Identification of miR signatures of Hematopoietic Stem Cells

**Figure 3:**
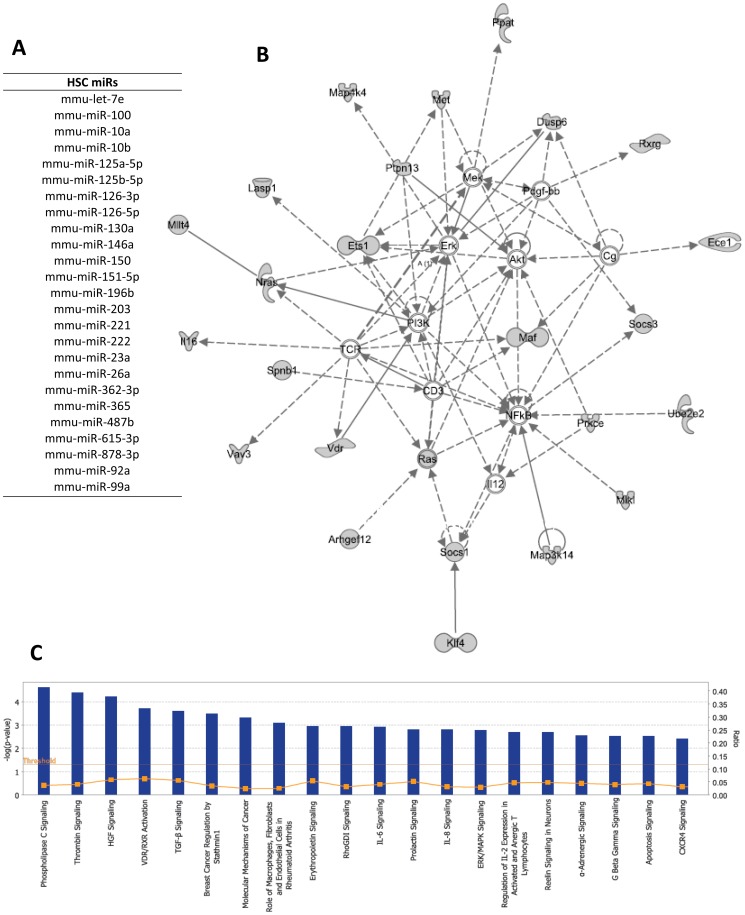
HSC-specific miRs target hematologic development. A) 25 “HSC miRs” are significantly overexpressed in HSC populations (LT-HSC, ST-HSC, MPP) as compared to more differentiated populations that have lost self-renewal and multipotency capacities (CMP, GMP, MEP) (p<0.05) B) A network composed of “HSC Targets”, predicted to be down-regulated by “HSC miRs”. This network is involved in cell-to-cell signaling, hematological development, and the inflammatory response. C) Top 20 enriched pathways amongst the HSC Targets group. Bars represent −log(p-value) as shown on left axis, while grey squares connected with a line represent the ratio of molecules in the pathway that were directly identified in the HSC Targets group (right axis).

We next sought to identify any miR signatures that were specific to early HSC subsets as opposed to more differentiated progenitor populations. Based on normalized array expression values, we compiled a list of miRs that were expressed significantly higher in the “HSC subsets” (LT-HSC, ST-HSC, and MPP) than in the more differentiated myeloid populations (CMP, GMP, and MEP) (p<0.05). We then removed all miRs that were not expressed at >100 copies per cell in any of these three HSC populations, using the “lenient” 95% confidence threshold described above. We also removed those where only 1 probe met the above criteria, to include only those miRs with at least 10% of probes significantly overexpressed in HSCs and expressed above 100 copies per cell. This resulted in a list of 25 “HSC miRs” that were over-represented and present at functional levels in mouse HSC subsets (Figure 3A). As expression of these “HSC miRs” is specific to the HSC subsets when compared with progenitors, we hypothesized that they may contribute to the phenotypes of self-renewal and multipotency maintained specifically in these subsets.

### Global network suppression by “HSC miRs”

To predict molecular mechanisms by which HSC miRs might be acting in a network to affect hematopoietic stem cell maintenance and self-renewal, we utilized our novel target prediction algorithm, allowing combination of expression data with sequence-based target prediction algorithms [13]. MiR-mRNA pairs were scored for inversely correlating expression patterns throughout HSPC development, as well as for their thermodynamically-predicted complementarity based on the TargetScan and MiRanda databases (Table S3) [17], [18]. To examine how our group of HSC miRs would affect cellular protein expression, we developed a list of predicted “HSC targets”. These targets consisted of mRNAs with statistically significant inverse patterns of expression, which also contained at least one predicted miR-binding site (Table 1). We next used Ingenuity software (www.ingenuity.com) to identify networks and pathways that might be affected by HSC miRs.

**Table 1:** Predicted targets of HSC miRS.

HSC Targets			
Trp53inp1	Dnmt3b	Arhgef12	Gprc5b
8430419L09Rik	Ociad2	Nfia	Maf
Spire1	C530008M17Rik	Cpm	Emid1
Trps1	Lasp1	Dusp6	Ppat
Antxr2	Lpp	Nras	Crim1
Ets1	Nsg1	Erg	Rxrg
Mllt6	Zfp608	Ece1	Gpsm1
Tacc2	Socs3	Itga9	Mlkl
Alpk3	B3gnt5	Il16	Pde10a
Srpk2	Fbxo21	Daam1	Cav2
Lhfpl2	Arhgef3	Fmnl2	Igh-VJ558
Dab2ip	Nfat5	Vdr	Nrgn
Map4k4	Bysl	Tmem120b	Myct1
Spsb4	Socs1	Coro2a	Tgfbr2
Meis1	Stxbp6	Epas1	Btaf1
Slc22a23	Cd2ap	Arhgef5	Zfp516
Rhoq	Runx2	Zfp827	Arpp21
Smad1	Map3k14	Spnb1	Tmem56
Met	Esr1	Paqr9	Klf4
5031439G07Rik	Plekha2	Sh3tc2	Col5a1
Sept8	Gusb	Epdr1	Ube2e2
Mast4	Hlf	Elk3	Atp10a
Limd2	Abcg1	Zfhx3	Mtpn
1500009L16Rik	Rab6b	Fbxo10	Vldlr
Spnb2	Atp8b4	Tpm3	B4galt4
Snn	Nrxn1	Mllt4	Ypel1
Hsd17b11	Sash1	Prkce	Ubac1
Vav3	Samd14	Gng2	Ptpn13
Aldh5a1	Adcy6	Cmtm3	

All targets are predicted by TargetScan and/or MiRanda to have one or more binding sites for at least one “HSC miR”. Additionally, each target shows a statistically significant inverse pattern of expression with its targeting miR, across our 6 HSPC populations (p<0.05).

The “HSC Target” group was composed of 115 transcripts predicted to be targets of “HSC miRs”. This group was significantly enriched for molecules involved in cancer (50) as well as in hematological malignancies (16) (p<0.05, right-tailed Fisher Exact Test). The top network identified was involved in cell-to-cell signaling and interaction, hematological system development and function, and inflammatory response (Figure 3B). As these attributes are essential factors in HSC biology, this gave us confidence that our algorithm was revealing miR-mRNA interactions with a functional role in HSCs. In addition, the HSC Target group was enriched for involvement in several canonical signaling pathways, the top 20 of which are shown in Figure 3C. The top enriched pathway, phospholipase C signaling, has been shown to be involved in maintaining HSC quiescence and controlling myeloid differentiation [19]. Phospholipase C gamma mediates cytokine signaling in both human and mouse HSPCs, and inhibition of this molecule prevents differentiation toward granulocyte/monocyte lineages [20]. In addition, the HSC Targets are enriched for cytokine signaling responses that might otherwise lead to differentiation – e.g., Erythropoietin, IL-2, IL-6, IL-8 signaling (Figure 3C).

### Identification of miRs with a myeloid differentiation signature

Next, to determine which miRs are candidates that might regulate granulocyte-monocyte and erythroid-megakaryocyte development, we examined those miRs that were highly expressed in GMP and MEP subsets, respectively. 12 miRs were expressed at significantly higher levels in GMPs than all of the other examined HSPC stages (p<0.05, one-sided t-test). We again limited this list to miRs with at least 2 probes (≥10% of total probes) significantly overexpressed, and those that were also expressed above our predicted functional threshold of 100 copies per cell (Table 2). This list includes miR-223, a known modulator of granulopoiesis [2]. Applying the same standards to the MEP population, 36 miRs were overexpressed (Table 2), including the miR-144∼451 cluster known to modulate erythroid development [3]. We also identified 14 “General Myeloid” miRs that were overexpressed in all of the myeloid progenitor subsets (CMP, GMP and MEP) as compared to the stem cell subsets (LT-HSC, ST-HSC, MPP) (Table 2).

**Table 2:** Myeloid-associated microRNAs.

GMP miRs	MEP miRs	General Myeloid miRs
mmu-miR-106b*	mmu-miR-1224	mmu-miR-101b
mmu-miR-139-5p	mmu-miR-134	mmu-miR-106a
mmu-miR-146b	mmu-miR-135a*	mmu-miR-133a
mmu-miR-148a	mmu-miR-144	mmu-miR-135a
mmu-miR-148b	mmu-miR-15b*	mmu-miR-141
mmu-miR-149	mmu-miR-185	mmu-miR-148a*
mmu-miR-15b*	mmu-miR-186	mmu-miR-17
mmu-miR-223	mmu-miR-18a	mmu-miR-191*
mmu-miR-27b	mmu-miR-290-5p	mmu-miR-19a*
mmu-miR-338-3p	mmu-miR-292-5p	mmu-miR-219
mmu-miR-340-3p	mmu-miR-30c-1*	mmu-miR-382*
mmu-miR-340-5p	mmu-miR-341	mmu-miR-425*
	mmu-miR-370	mmu-miR-671-3p
	mmu-miR-378	mmu-miR-93
	mmu-miR-451	
	mmu-miR-452	
	mmu-miR-466c-5p	
	mmu-miR-483	
	mmu-miR-486	
	mmu-miR-574-5p	
	mmu-miR-669c	
	mmu-miR-681	
	mmu-miR-682	
	mmu-miR-691	
	mmu-miR-696	
	mmu-miR-703	
	mmu-miR-705	
	mmu-miR-710	
	mmu-miR-711	
	mmu-miR-712	
	mmu-miR-712*	
	mmu-miR-714	
	mmu-miR-721	
	mmu-miR-762	
	mmu-miR-7a	
	mmu-miR-805	

A) 12 “GMP miRs” are significantly overexpressed in the GMP population compared to all other populations profiled (p<0.05). 36 “MEP miRs” are significantly overexpressed in the MEP population, and 14 “General Myeloid miRs” are overexpressed in all three myeloid populations (CMP, GMP, MEP) as compared to less differentiated stem-progenitor populations (LT-HSC, ST-HSC, MPP). All miRs listed had at least 2 probes (≥10% of total probes) with significant overexpression and an absolute copy number level above our predicted functional threshold.

To predict how these differentiation-specific miRs might be acting in a network to affect mono/granulopoiesis, we utilized our algorithm to predict which targets these groups of miRs may regulate. The predicted target network regulated by “GMP miRs” was enriched for both cancer and hematological disease genes, as well as those involved in hematological development/function, and hematopoiesis (p<0.05, right-tailed Fisher Exact Test) (Table S4). This was also true for the network of targets predicted for “MEP miRs” (p<0.05) (Table S5). Surprisingly, in contrast to the other groups examined, our analysis of the “General Myeloid miRs” group produced zero significant targets (discussed below).

### Validation of Target Prediction Algorithm

**Figure 4:**
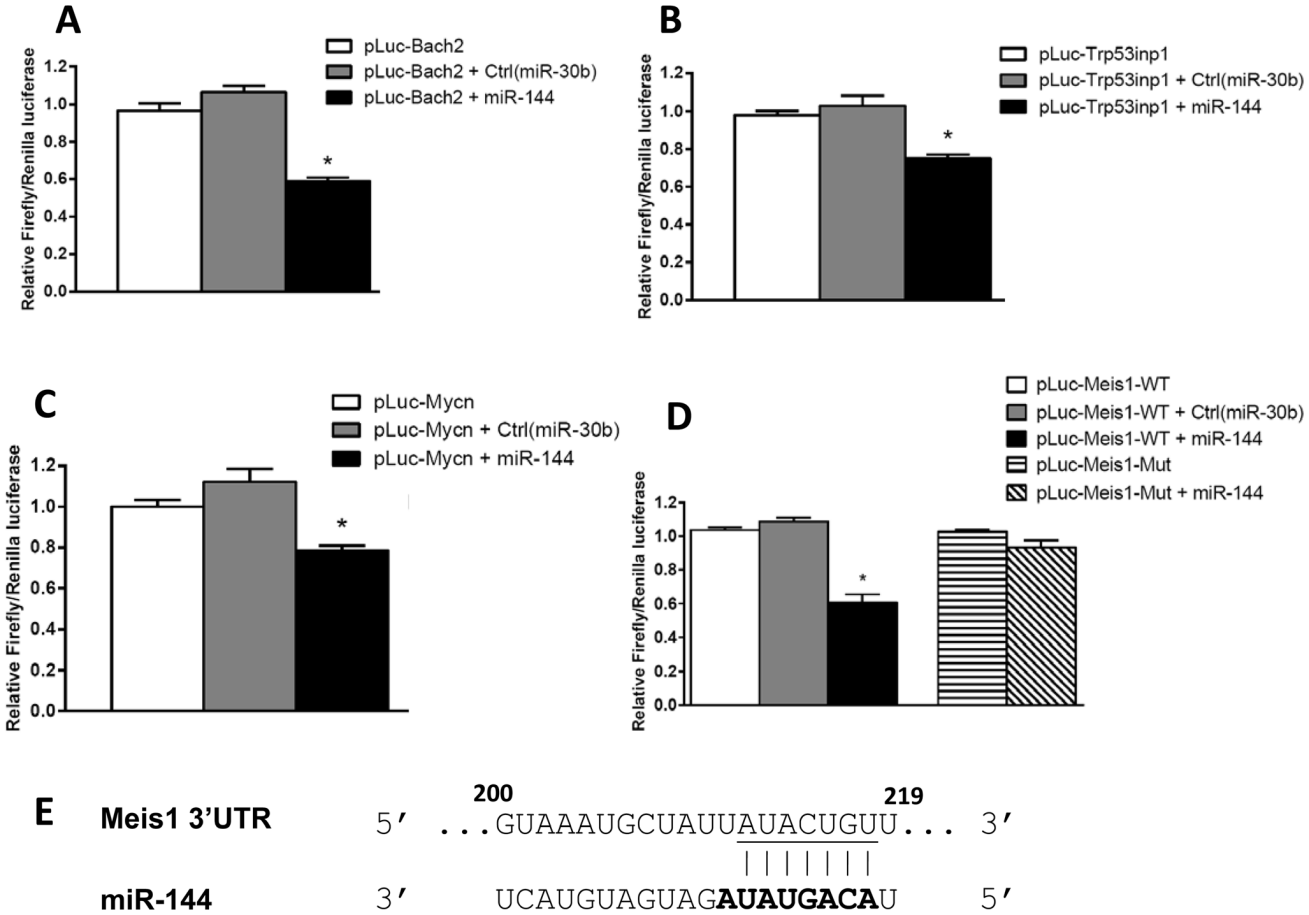
Luciferase assay validation of 4 top predicted candidates. HEK293T cells were transfected with pLuc-3′UTR plasmids with or without the indicated miR mimics, and cell lysates were harvested for luciferase assay 24 hrs after transfection. MiR-144 suppressed luciferase activity through the Bach2 (A), Trp53inp1 (B), and MycN(C) 3′UTR sequences, whereas control miR-30b resulted in no luciferase repression in all cases. (D) MiR-144 also suppressed luciferase activity through the Meis1 3′UTR sequence. The miR-144 binding site in the Meis1-3′UTR was further confirmed by testing a mutant Meis1 3′UTR construct (pLuc-Meis-Mut) in which the predicted miR-144 seed sequence was deleted. Panel E depicts the miR-144 predicted binding site in the Meis1 3′UTR (bases 200–219 of the 3′UTR are shown). The underlined sequence denotes the nucleotides deleted in the mutant Meis1 construct, with the bolded nucleotides in the mature miR-144 sequence indicating the miR-144 seed sequence. Deletion of these nucleotides abrogated the repression of luciferase activity by miR-144. In all panels, Firefly luciferase activity was normalized to Renilla luciferase activity, and data shown is relative to cells transfected with only the pLuc-3′UTR plasmids. (Means±SEM, unpaired Student *t* test, n≥3) Statistical significance is denoted by *P<0.05 in all panels.

We tested four of the top predicted targets of miR-144 in luciferase assays, in order to determine whether they were actual direct targets. All four 3′ UTRs were significantly down-regulated in the presence of miR-144, but not in the presence of a control miR (Figure 4A–D). We performed site-directed mutagenesis on the Meis1 3′UTR to delete the predicted miR-144 target site. Suppression of luciferase activity by miR-144 was ablated, indicating that miR-144 directly targets the Meis1 3′UTR via the predicted target sequence (Figure 4D). While validation of a wider subset of predicted interactions should be performed moving forward with the dataset, the validation of all 4 current candidates that we chose for testing lends confidence to the accuracy of our database of predicted miR-mRNA interactions.

## Discussion

Herein, we attempt to provide a compendium of miR expression during early hematopoiesis. We examine our data through a lens that includes both absolute expression levels for miRs, as well as potential miR-mRNA interactions. By providing estimates of absolute quantification, as opposed to normalized array values only, we attempt to provide a view into which miRs are most likely to be functioning in HSPC subsets.

Based on current reports [10], [11], [12], we set our functional threshold at 100 copies per cell. Although it is not possible to correlate array value with absolute copy number exactly (as this will vary for different miR probes), we provided an estimated value based on a regression analysis of a set of 15 miRs. In order to attain 95% confidence in identifying all miRs that are expressed above 100 copies per cell (p<0.05), we included miRs that may be slightly below this level. In addition, by using 8 pg/cell RNA content (the highest measured in any of our HSPC populations) we have again erred on the side of inclusion. While we have chosen to be inclusive in our analysis of the data, the threshold we have set can be changed by investigators querying our datasets (GEO dataset GSE49170) to be more or less stringent. We believe that our candidate lists identify the most promising candidate miRs and target molecules, and we have utilized them to evaluate how miRs may be functioning together in a network regulating early hematopoiesis.

We have also used a novel Bayesian model to improve the analysis of predicted miR-mRNA interactions [13]. As with all models of small-sample RNA expression data, the number of variables (RNA expression values and interactions) inferred far exceeds the number of samples available. Thus, the results are susceptible to random noise causing false correlation, but multiple-testing correction and the propagation of variance and certainty throughout the Bayesian model can mitigate this risk. Therefore, false positives are always possible, if not expected, amongst the most significant findings, though the incorporation of sequence-based target prediction databases along with the expression data should enrich the results to contain a higher proportion of true positives than results from either data type by itself.

Our data identifies virtually all known miR modulators of early hematopoiesis that have been reported previously. This includes the clustered miR-144 and miR-451 as modulators of erythropoiesis, miR-223 as a modulator of granulopoiesis, and family members miR-125a/125b as modulators of hematopoietic stem cell proliferation and maintenance [2], [3], [7], [21]. It is also promising that predicted target lists from HSC miRs and early myeloid miRs were significantly enriched in molecules and networks involved in hematopoietic development (Figure 3). The fact that these targets were also enriched in cancer and hematological malignancy pathways points to the link between cancer and stem cell pathways, and suggests these “HSC miRs” as potential regulators of these processes.

It is interesting to note that our algorithm uncovered no significant miR-mRNA target pairs for “General Myeloid miRs”. While the “GMP miRs” list was composed of 12 miRs predicted to target 242 genes, the list of “General myeloid miRs”, composed of 14 miRs, unexpectedly had no predicted targets. While we did not expect this result, it may reflect a relatively minor contribution of miRs at this stage in hematopoietic development, as compared to stem cell populations and differentiation further downstream. In fact, in a recent publication where all miRs were knocked out in the earliest myeloid-specific progenitors, there were no differences observed in CMP, GMP or MEP numbers or function. Defects were instead observed downstream, as neutrophils were unable to mature into functional immune cells and monocyte/macrophage development was perturbed [22]. It may also be possible that the important targets of the “General Myeloid miRs” are regulated at the level of translational repression rather than at the mRNA level. Indeed, this is a general limitation of our algorithm at all stages of hematopoietic differentiation, as the algorithm relies solely on mRNA (as opposed to protein) levels for target prediction.

Interestingly, we note that across our 6 examined populations which included both stem cell populations and early myeloid populations, HSPC miR expression was quite similar. Nearly half of all miR families expressed in HSPCs (n = 46) were present in all six populations, while only 14 families were unique to one distinct population (Table S2). Our data aligns well with studies from other labs that have reported similar miR expression data. The Scadden group profiled LT-HSC, ST-HSC, and MPP populations using a bead-based array platform and described 16 miRs that are upregulated in these self-renewal-competent populations [7]. Of these, 9 overlap with our generated list of HSC miRs, and an additional 2 are from families represented in our list. Our data also correlates well with a qPCR-based profiling study of 288 miRs throughout hematopoiesis [23]. In this study, 5 out of 7 miRs found to be over-represented in HSC subsets are also found in our “HSC miR” group, or are represented by family members in this group. Our data also overlaps considerably with several studies profiling subsets of human HSPCs [24] (Civin lab, unpublished data).

A distinct advantage to the approach of our current study is the profiling of each sequential progenitor subset. With this data, we are able to compare HSC subpopulations with each individual stage of differentiation, as opposed to a general pool of HSPCs or whole bone marrow. This allows for a finer discrimination of which miRs are expressed only in populations with extensive self-renewal capacity, as well as the construction of a detailed map of the precise stage of differentiation at which any given miR is “turned on” or off. In addition, the ordered nature of our samples lent more power to the statistical analysis of miR-mRNA pattern correlations, allowing further discrimination in predicted mRNA targets as well [13]. Our predictions were validated in all 4 of the candidate miR-mRNA interactions chosen for biological testing using a luciferase assay. This suggests that our novel interaction database may contain a substantially lower false positive rate than previously attained, and lends strength to the predictions listed. Additionally, by compiling a global view of miR-mRNA interactions during early hematopoiesis, this database makes it possible to investigate broad consequences of multiple miRs functioning simultaneously throughout the transition from stem cells to differentiated progenitors. For example, the database provides a resource for examining the effect of microRNA in controlling phospholipase C signaling in HSCs, rather than examining the effect of a single miR on a single signaling molecule [19].

To our knowledge this is the first array-based database compiled contrasting miR and mRNA expression in each individual population of early HSPC subsets. We have used this novel database to create an extensive map of global miR networks, and further to predict mRNA targets of these miR networks. The database described here provides several promising candidates for functional validation, and we hope it will serve as a valuable resource for others studying miR function during early hematopoiesis.

## Materials and Methods

### Ethics Statement

This study and all protocols utilized in the study were approved by the University of Maryland School of Medicine IACUC (Protocol #1111004).

### Mice

All mice used in these experiments were female 6–8 week old C57Bl/6 (Jackson Labs). 10–20 mice were pooled for each replicate sample.

### Bone Marrow Isolation and Enrichment

Bone marrow was harvested by crushing tibias, femurs, hips and spine of each mouse with a mortar and pestle as previously described [25]. Lineage depletion was performed using the following biotin-conjugated antibodies; anti-B220, CD3, GR-1, Ter119 (BD Bioscience). Bone marrow was then incubated with anti-biotin microbeads and depleted using XS columns on a SuperMACS magnet (Miltenyi). Lin-depleted cells were enriched for c-kit-expressing cells using APC-conjugated anti-c-kit (BD bioscience) followed by anti-APC microbeads for positive selection on LS magnetic columns (Miltenyi). Magnetic separations were performed as per manufacturer's instructions.

### Flow cytometry and cell sorting

Sorting of Lin-/kit+ hematopoietic subpopulations was conducted on a FACS Aria (BD Biosciences) and analyzed using FlowJo software (Flow Jo). Antibodies used to separate HSPC populations were as follows: streptavidin-PerCP-Cy5.5, c-kit-APC, FLT3-PE, and FcRγ-PE from BD Bioscience (San Jose, CA), and sca-1-PE-Cy7 and CD34-FITC from ebioscience (San Diego, CA).

### RNA preparation and microarray hybridization

RNA was isolated from Trizol using a miRNeasy Micro kit according to manufacturer's instructions (Qiagen). RNA was labeled and hybridized to single-color Agilent Mouse MicroRNA Arrays (Version 1) and to Agilent single color Whole Genome 4×44k Mouse mRNA arrays at Cogenics-Beckman Coulter facility (Morrisville, NC).

### Microarray Data Analysis

Analysis of microarray data for both miR and mRNA profiling was done using the *limma* [26], [27] package of *R* [28]. The data were normalized via the “quantile” method, independently for miR and mRNA data, and the functions lmFit() and ebayes() were used to fit a linear model and perform all statistical tests used, with the exception of the miR-mRNA targeting analysis. All data has been deposited in the GEO database – dataset GSE49170 ().

### qPCR and absolute miR quantification

miR qPCR was performed using a Taqman RT kit (7.5 ul total reaction) and qPCR primers (10 ul total reaction) as per manufacturer's protocol (Applied Biosystems). RNA oligonucleotides synthesized by IDT and corresponding to the mature miR sequence (miRbase.org) were used to create standard curves for absolute quantification.

### miR-mRNA targeting analysis

We used the model described in [13] to infer targeting interactions between miRs and mRNAs. In short, this model, written in R [28] is a Bayesian network of miR and mRNA expression values in which a partial ordering of the samples/stages is utilized to infer the most likely interactions by simultaneously comparing expression profiles from all miRs and mRNAs. The model also includes target prediction information from both TargetScan [17] and miRanda/microrna.org [18] allowing (but not forcing) the model to use these sequence-based predictions to improve target pair inference. The output from these calculations is a table of miR-mRNA pairs, ranked by the statistical significance of the inferred interaction. Because of the considerable run time required by the algorithm, we included in these analyses only those miRs and mRNAs with the most significant F-test p-values, when testing for any differential expression across stages. We found that p<10^−6^ sufficiently reduced the data by removing probes whose expression changed relatively little across sample stages, while leaving many highly active miRs and mRNAs. Finally, we left out those miRs and mRNAs for which no target predictions exist in either TargetScan or miRanda, leaving a total of 64 miRs and 261 mRNAs as input for the target pair inference algorithm.

### Target Network Analysis

Analyses for functional and network enrichment in predicted miR target lists were generated using IPA software (Ingenuity Systems, www.ingenuity.com).

### Luciferase Assays

cDNA was amplified from whole bone marrow harvested from a wild-type C57/Bl6 mouse using SuperScriptIII with random hexamer primers, according to manufacturer's instructions (Invitrogen). Primers used for subsequent specific PCR amplification were designed according to 3′UTR sequences provided by NCBI, and UTR sequences were cloned at least 200 base pairs upstream and downstream from predicted target sites. For the luciferase assay, HEK293T cells were cotransfected in 24-well plates using Lipofectamine 2000 (Life Technologies-Invitrogen) according to the manufacturer's protocol, with 100 ng Firefly luciferase reporter plasmid (pcDNA3.1-Luc-3′UTR, referred to as pLuc) and 25 ng control vector containing Renilla luciferase pRL-TK Vector (Promega). 50 nM mmu-miR-144-3p or mmu-miR-30b-5p miRIDIAN miR mimics were used in the assays (Thermo Scientific Dharmacon). Luciferase assays were performed 24 hrs after transfection using the Dual-Luciferase Reporter Assay System (Promega). Firefly luciferase activity in each well was normalized to Renilla luciferase activity, and data is shown relative to the empty pLuc construct backbone. Site-directed mutagenesis to delete the miR-144 binding site in the Meis1-3′UTR was performed using the QuikChange Lightning Site-Directed Mutagenesis kit according to the manufacturer's protocol (Agilent Technologies). Unpaired student's t-test was performed for statistical analysis (n≥3).

## Supporting Information

Table S1 **Individual miRs expressed over the threshold in HSPC populations.** A) miRs expressed over 100 copies per cell in the LT-HSC Population B) miRs expressed over 100 copies per cell in the ST-HSC Population C) miRs expressed over 100 copies per cell in the MPP Population D) miRs expressed over 100 copies per cell in the CMP Population E) miRs expressed over 100 copies per cell in the GMP Population F) miRs expressed over 100 copies per cell in the MEP Population
(DOCX)

Table S2 **miR family expression in HSPC subsets.** miRs are grouped by family, and HSPC populations expressing each family over 100 copies per cell are listed. ALL indicates that the miR family is represented over the threshold in all HSPC populations examined (LT-HSC, ST-HSC, MPP, CMP, GMP and MEP). If the miR is not a member of a known family, the family listed refers to the individual microRNA itself (mmu-miR-xx)
(DOCX)

Table S3 **miR-mRNA predicted target pairs.** miR-mRNA pairs were scored for inversely correlating expression patterns throughout hematopoietic progenitor development, as well as for their thermodynamically-predicted complementarity based on the TargetScan and MiRanda databases.
(XLSX)

Table S4 **Predicted targets of GMP miRs.** 242 GMP targets predicted by TargetScan and/or MiRanda to have binding sites for at least one “GMP miR”. Additionally, each target shows a statistically significant inverse pattern of expression with its targeting miR, across our 6 HSPC populations (p<0.05).
(DOCX)

Table S5 **Predicted Targets of MEP miRs.** 55 targets predicted by TargetScan and/or MiRanda to have binding sites for at least one “MEP miR”. Additionally, each target shows a statistically significant inverse pattern of expression with its targeting miR, across our 6 HSPC populations (p<0.05).
(DOCX)
