# Supplementary material for: Correlated miR-mRNA Expression Signatures of Mouse Hematopoietic Stem and Progenitor Cell Subsets Predict “Stemness” and “Myeloid” Interaction Networks
Source: PLoS One. 2014 Apr 18;9(4):e94852. doi: 10.1371/journal.pone.0094852 (PMC3991639; doi:10.1371/journal.pone.0094852)
Supplement: Table S1 — Individual miRs expressed over the threshold in HSPC populations. A) miRs expressed over 100 copies per cell in the LT-HSC Population B) miRs expressed over 100 copies per cell in the ST-HSC Population C) miRs expressed over 100 copies per cell in the MPP Population D) miRs expressed over 100 copies per cell in the CMP Population E) miRs expressed over 100 copies per cell in the GMP Population F) miRs expressed over 100 copies per cell in the MEP Population (DOCX) [file pone.0094852.s001.docx]

**Table S1: Individual miRs expressed over the threshold in HSPC populations**

Table S1a: miRs expressed over 100 copies per cell in the LT-HSC Population

| **miR** | **Family** | **Normalized Array Intensity** |
| --- | --- | --- |
| mmu-miR-720 | miR-720.m | 13.64571 |
| mmu-miR-1224 | miR-1224-5p/1671 | 12.12545 |
| mmu-miR-142-3p | miR-142-3p | 11.67633 |
| mmu-miR-690 | miR-690 | 11.612 |
| mmu-miR-494 | miR-494 | 11.33686 |
| mmu-miR-21 | miR-21/590-5p | 11.17666 |
| mmu-miR-92a | miR-25/32/92abc/363/363-3p/367 | 11.14099 |
| mmu-miR-19b | miR-19ab | 11.07376 |
| mmu-miR-689 | none | 10.89855 |
| mmu-miR-451 | miR-451 | 10.7519 |
| mmu-miR-709 | miR-709/1827 | 10.62261 |
| mmu-miR-26a | miR-26ab/1297/4465 | 10.34545 |
| mmu-miR-20a | miR-17/17-5p/20ab/20b-5p/93/106ab/427/518a-3p/519d | 10.33781 |
| mmu-miR-16 | miR-15abc/16/16abc/195/322/424/497/1907 | 10.31803 |
| mmu-miR-29a | miR-29abcd | 10.20213 |
| mmu-miR-15b | miR-15abc/16/16abc/195/322/424/497/1907 | 10.19439 |
| mmu-let-7i | let-7/98/4458/4500 | 10.1827 |
| mmu-miR-223 | miR-223 | 10.06619 |
| mmu-let-7a | let-7/98/4458/4500 | 9.864644 |
| mmu-miR-148a | miR-148ab-3p/152 | 9.83495 |
| mmu-let-7f | let-7/98/4458/4500 | 9.821662 |
| mmu-miR-130a | miR-130ac/301ab/301b/301b-3p/454/721/4295/3666 | 9.802686 |
| mmu-let-7g | let-7/98/4458/4500 | 9.470956 |
| mmu-miR-15a | miR-15abc/16/16abc/195/322/424/497/1907 | 9.417201 |
| mmu-miR-23a | miR-23abc/23b-3p | 9.340492 |
| mmu-miR-20b | miR-17/17-5p/20ab/20b-5p/93/106ab/427/518a-3p/519d | 9.324103 |
| mmu-miR-29b | miR-29abcd | 9.275655 |
| mmu-miR-19a | miR-19ab | 9.090995 |
| mmu-miR-106b | miR-17/17-5p/20ab/20b-5p/93/106ab/427/518a-3p/519d | 9.054403 |
| mmu-miR-714 | miR-714 | 9.026313 |
| mmu-miR-24 | miR-24/24ab/24-3p | 8.956783 |
| mmu-miR-25 | miR-25/32/92abc/363/363-3p/367 | 8.85739 |
| mmu-let-7d | let-7/98/4458/4500 | 8.82965 |
| mmu-miR-142-5p | miR-142-5p | 8.786986 |
| mmu-miR-107 | miR-103a/107/107ab | 8.774618 |
| mmu-miR-370 | miR-370 | 8.658091 |
| mmu-miR-574-5p | miR-574-5p | 8.63693 |
| mmu-miR-93 | miR-17/17-5p/20ab/20b-5p/93/106ab/427/518a-3p/519d | 8.609456 |
| mmu-miR-125b-5p | miR-125a-5p/125b-5p/351/670/4319 | 8.580202 |
| mmu-let-7c | let-7/98/4458/4500 | 8.511405 |
| mmu-miR-212 | none | 8.509947 |
| mmu-miR-26b | miR-26ab/1297/4465 | 8.48789 |
| mmu-miR-30c | miR-30abcdef/30abe-5p/384-5p | 8.458466 |
| mmu-miR-202-3p | miR-202-3p | 8.43926 |
| mmu-miR-10a | miR-10abc/10a-5p | 8.358695 |
| mmu-miR-30d | miR-30abcdef/30abe-5p/384-5p | 8.269692 |
| mmu-miR-30b | miR-30abcdef/30abe-5p/384-5p | 8.264968 |
| mmu-let-7b | let-7/98/4458/4500 | 8.094998 |
| mmu-miR-721 | miR-130ac/301ab/301b/301b-3p/454/721/4295/3666 | 8.033261 |
| mmu-miR-103 | miR-103a/107/107ab | 8.013878 |
| mmu-miR-146a | miR-146ac/146b-5p | 8.010249 |
| mmu-miR-181a | miR-181abcd/4262 | 7.945056 |
| mmu-miR-126-3p | miR-126-3p | 7.914151 |
| mmu-miR-652 | miR-652 | 7.903102 |
| mmu-miR-30e | miR-30abcdef/30abe-5p/384-5p | 7.813398 |
| mmu-miR-680 | miR-680 | 7.785551 |
| mmu-miR-27a | miR-27abc/27a-3p | 7.7489 |
| mmu-miR-140* | none | 7.722408 |
| mmu-miR-18a | miR-18ab/4735-3p | 7.686499 |
| mmu-miR-99a | miR-99ab/100 | 7.578911 |
| mmu-miR-671-5p | miR-671-5p | 7.542619 |
| mmu-miR-23b | miR-23abc/23b-3p | 7.532842 |
| mmu-miR-29c | miR-29abcd | 7.473382 |
| mmu-miR-188-5p | miR-188-5p | 7.458218 |
| mmu-miR-17 | miR-17/17-5p/20ab/20b-5p/93/106ab/427/518a-3p/519d | 7.448302 |
| mmu-miR-22 | miR-22/22-3p | 7.443397 |
| mmu-miR-425 | miR-425/425-5p/489 | 7.40871 |
| mmu-miR-130b | miR-130ac/301ab/301b/301b-3p/454/721/4295/3666 | 7.306284 |
| mmu-miR-801 | none | 7.299159 |
| mmu-miR-322 | miR-15abc/16/16abc/195/322/424/497/1907 | 7.284253 |
| mmu-miR-106a | miR-17/17-5p/20ab/20b-5p/93/106ab/427/518a-3p/519d | 7.280708 |
| mmu-miR-181c | miR-181abcd/4262 | 7.247954 |
| mmu-miR-483 | miR-483-5p | 7.220724 |
| mmu-miR-222 | miR-221/222/222ab/1928 | 7.13886 |
| mmu-miR-221 | miR-221/222/222ab/1928 | 7.041509 |
| mmu-miR-17* | 17* | 7.00355 |
| mmu-miR-34a | miR-34ac/34bc-5p/449abc/449c-5p | 6.90804 |
| mmu-miR-712 | miR-712 | 6.855904 |
| mmu-miR-486 | miR-486-5p/3107 | 6.827585 |
| mmu-miR-705 | miR-705/2897 | 6.816265 |
| mmu-miR-181b | miR-181abcd/4262 | 6.790855 |
| mmu-miR-378 | miR-378/422a/378bcdefhi | 6.781945 |
| mmu-miR-196b | miR-196abc | 6.62735 |
| mmu-miR-135a* | 135a* | 6.540326 |
| mmu-miR-706 | miR-706 | 6.53213 |
| mmu-miR-181d | miR-181abcd/4262 | 6.525139 |
| mmu-miR-345-5p | miR-345-5p.m | 6.524498 |
| mmu-miR-669c | miR-669c | 6.521291 |
| mmu-miR-27b | miR-27abc/27a-3p | 6.500978 |
| mmu-miR-139-3p | miR-139-3p.dmr | 6.498012 |
| mmu-miR-374 | miR-374ab | 6.473277 |
| mmu-miR-101a | miR-101/101ab | 6.452801 |
| mmu-miR-30a | miR-30abcdef/30abe-5p/384-5p | 6.445281 |
| mmu-miR-140 | miR-140/140-5p/876-3p/1244 | 6.441469 |
| mmu-miR-150 | miR-150/5127 | 6.406591 |
| mmu-let-7e | let-7/98/4458/4500 | 6.404018 |
| mmu-miR-762 | miR-762/4492/4498 | 6.402479 |
| mmu-miR-697 | miR-697 | 6.389594 |
| mmu-miR-125a-3p | miR-125a-3p/1554 | 6.387529 |
| mmu-miR-146b | miR-146ac/146b-5p | 6.385377 |
| mmu-miR-144 | miR-144 | 6.346576 |
| mmu-miR-290-5p | miR-290-5p/292-5p/371-5p/293 | 6.343988 |
| mmu-miR-155 | miR-155 | 6.321749 |
| mmu-miR-101b | miR-101/101ab | 6.321312 |
| mmu-miR-320 | miR-320abcd/4429 | 6.318112 |
| mmu-miR-301a | miR-130ac/301ab/301b/301b-3p/454/721/4295/3666 | 6.317561 |
| mmu-miR-341 | miR-341 | 6.306722 |
| mmu-miR-877* | none | 6.297008 |

Table S1b: miRs expressed over 100 copies per cell in the ST-HSC Population

| **miR** | **Family** | **Normalized Array Intensity** |
| --- | --- | --- |
| mmu-miR-720 | miR-720.m | 12.38848 |
| mmu-miR-19b | miR-19ab | 11.92203 |
| mmu-miR-21 | miR-21/590-5p | 11.73396 |
| mmu-miR-142-3p | miR-142-3p | 11.45354 |
| mmu-miR-223 | miR-223 | 11.2633 |
| mmu-miR-92a | miR-25/32/92abc/363/363-3p/367 | 11.17678 |
| mmu-let-7f | let-7/98/4458/4500 | 11.06522 |
| mmu-miR-15b | miR-15abc/16/16abc/195/322/424/497/1907 | 10.86133 |
| mmu-miR-1224 | miR-1224-5p/1671 | 10.79619 |
| mmu-let-7a | let-7/98/4458/4500 | 10.7959 |
| mmu-miR-709 | miR-709/1827 | 10.67494 |
| mmu-miR-20a | miR-17/17-5p/20ab/20b-5p/93/106ab/427/518a-3p/519d | 10.55962 |
| mmu-miR-26a | miR-26ab/1297/4465 | 10.12976 |
| mmu-miR-106b | miR-17/17-5p/20ab/20b-5p/93/106ab/427/518a-3p/519d | 10.12314 |
| mmu-miR-689 | none | 10.03346 |
| mmu-miR-19a | miR-19ab | 9.988202 |
| mmu-let-7g | let-7/98/4458/4500 | 9.943559 |
| mmu-let-7i | let-7/98/4458/4500 | 9.905324 |
| mmu-miR-23a | miR-23abc/23b-3p | 9.88726 |
| mmu-let-7d | let-7/98/4458/4500 | 9.855108 |
| mmu-miR-20b | miR-17/17-5p/20ab/20b-5p/93/106ab/427/518a-3p/519d | 9.740452 |
| mmu-miR-29a | miR-29abcd | 9.665264 |
| mmu-miR-690 | miR-690 | 9.643786 |
| mmu-miR-494 | miR-494 | 9.534794 |
| mmu-miR-26b | miR-26ab/1297/4465 | 9.492788 |
| mmu-miR-25 | miR-25/32/92abc/363/363-3p/367 | 9.475622 |
| mmu-let-7c | let-7/98/4458/4500 | 9.416416 |
| mmu-miR-107 | miR-103a/107/107ab | 9.276258 |
| mmu-miR-15a | miR-15abc/16/16abc/195/322/424/497/1907 | 9.211132 |
| mmu-miR-130a | miR-130ac/301ab/301b/301b-3p/454/721/4295/3666 | 9.197426 |
| mmu-miR-16 | miR-15abc/16/16abc/195/322/424/497/1907 | 9.128402 |
| mmu-let-7b | let-7/98/4458/4500 | 9.11527 |
| mmu-miR-142-5p | miR-142-5p | 9.024952 |
| mmu-miR-24 | miR-24/24ab/24-3p | 8.931449 |
| mmu-miR-27a | miR-27abc/27a-3p | 8.832488 |
| mmu-miR-30c | miR-30abcdef/30abe-5p/384-5p | 8.707145 |
| mmu-miR-221 | miR-221/222/222ab/1928 | 8.701941 |
| mmu-miR-30b | miR-30abcdef/30abe-5p/384-5p | 8.688504 |
| mmu-miR-23b | miR-23abc/23b-3p | 8.687548 |
| mmu-miR-10a | miR-10abc/10a-5p | 8.667962 |
| mmu-miR-202-3p | miR-202-3p | 8.463103 |
| mmu-miR-322 | miR-15abc/16/16abc/195/322/424/497/1907 | 8.456414 |
| mmu-miR-103 | miR-103a/107/107ab | 8.433138 |
| mmu-miR-29b | miR-29abcd | 8.218361 |
| mmu-miR-30d | miR-30abcdef/30abe-5p/384-5p | 8.129604 |
| mmu-miR-93 | miR-17/17-5p/20ab/20b-5p/93/106ab/427/518a-3p/519d | 8.120648 |
| mmu-miR-148a | miR-148ab-3p/152 | 8.072581 |
| mmu-miR-125b-5p | miR-125a-5p/125b-5p/351/670/4319 | 8.048546 |
| mmu-miR-30e | miR-30abcdef/30abe-5p/384-5p | 8.014075 |
| mmu-miR-18a | miR-18ab/4735-3p | 8.006469 |
| mmu-miR-222 | miR-221/222/222ab/1928 | 7.830902 |
| mmu-miR-181a | miR-181abcd/4262 | 7.792018 |
| mmu-miR-99a | miR-99ab/100 | 7.773216 |
| mmu-miR-140* | none | 7.728933 |
| mmu-miR-425 | miR-425/425-5p/489 | 7.685452 |
| mmu-miR-130b | miR-130ac/301ab/301b/301b-3p/454/721/4295/3666 | 7.658419 |
| mmu-miR-212 | none | 7.512385 |
| mmu-miR-181c | miR-181abcd/4262 | 7.510642 |
| mmu-miR-451 | miR-451 | 7.493861 |
| mmu-miR-196b | miR-196abc | 7.489531 |
| mmu-miR-370 | miR-370 | 7.446676 |
| mmu-miR-671-5p | miR-671-5p | 7.392522 |
| mmu-miR-714 | miR-714 | 7.358224 |
| mmu-miR-340-5p | miR-340-5p | 7.357558 |
| mmu-miR-301a | miR-130ac/301ab/301b/301b-3p/454/721/4295/3666 | 7.342653 |
| mmu-miR-721 | miR-130ac/301ab/301b/301b-3p/454/721/4295/3666 | 7.316956 |
| mmu-miR-574-5p | miR-574-5p | 7.311679 |
| mmu-miR-17 | miR-17/17-5p/20ab/20b-5p/93/106ab/427/518a-3p/519d | 7.28742 |
| mmu-miR-126-3p | miR-126-3p | 7.268006 |
| mmu-miR-17* | none | 7.2669 |
| mmu-miR-374 | miR-374ab | 7.22994 |
| mmu-miR-106a | miR-17/17-5p/20ab/20b-5p/93/106ab/427/518a-3p/519d | 7.195047 |
| mmu-miR-27b | miR-27abc/27a-3p | 7.176134 |
| mmu-miR-29c | miR-29abcd | 7.171366 |
| mmu-miR-652 | miR-652 | 7.110205 |
| mmu-miR-680 | miR-680 | 7.064399 |
| mmu-miR-450a-5p | none | 7.01833 |
| mmu-miR-188-5p | miR-188-5p | 6.961729 |
| mmu-miR-801 | none | 6.953639 |
| mmu-miR-181b | miR-181abcd/4262 | 6.941004 |
| mmu-miR-101a | miR-101/101ab | 6.869759 |
| mmu-miR-101b | miR-101/101ab | 6.857024 |
| mmu-miR-155 | miR-155 | 6.836066 |
| mmu-miR-712 | miR-712 | 6.82384 |
| mmu-miR-146b | miR-146ac/146b-5p | 6.822009 |
| mmu-miR-350 | miR-350 | 6.804194 |
| mmu-miR-98 | let-7/98/4458/4500 | 6.796193 |
| mmu-let-7e | let-7/98/4458/4500 | 6.769661 |
| mmu-miR-697 | miR-697 | 6.763071 |
| mmu-miR-181d | miR-181abcd/4262 | 6.712225 |
| mmu-miR-487b | miR-487b | 6.660758 |
| mmu-miR-378 | miR-378/422a/378bcdefhi | 6.646677 |
| mmu-miR-140 | miR-140/140-5p/876-3p/1244 | 6.646661 |
| mmu-miR-483 | miR-483-5p | 6.595138 |
| mmu-miR-34a | miR-34ac/34bc-5p/449abc/449c-5p | 6.543168 |
| mmu-miR-30a | miR-30abcdef/30abe-5p/384-5p | 6.527469 |
| mmu-miR-345-5p | miR-345-5p.m | 6.527382 |
| mmu-miR-146a | miR-146ac/146b-5p | 6.513596 |
| mmu-miR-195 | miR-15abc/16/16abc/195/322/424/497/1907 | 6.447401 |
| mmu-miR-203 | miR-203 | 6.394935 |
| mmu-miR-324-3p | miR-324-3p | 6.316055 |
| mmu-miR-128 | miR-128/128ab | 6.286374 |
| mmu-miR-705 | miR-705/2897 | 6.283672 |
| mmu-miR-125a-3p | miR-125a-3p/1554 | 6.283658 |
| mmu-miR-219 | none | 6.270092 |
| mmu-miR-139-3p | miR-139-3p.dmr | 6.263534 |
| mmu-miR-320 | miR-320abcd/4429 | 6.252149 |

Table S1c: miRs expressed over 100 copies per cell in the MPP Population

| **miR** | **Family** | **Normalized Array Intensity** |
| --- | --- | --- |
| mmu-miR-720 | miR-720.m | 12.33008 |
| mmu-miR-19b | miR-19ab | 12.13191 |
| mmu-let-7f | let-7/98/4458/4500 | 11.71442 |
| mmu-let-7a | let-7/98/4458/4500 | 11.61516 |
| mmu-miR-92a | miR-25/32/92abc/363/363-3p/367 | 11.56585 |
| mmu-miR-21 | miR-21/590-5p | 11.23807 |
| mmu-miR-15b | miR-15abc/16/16abc/195/322/424/497/1907 | 10.96537 |
| mmu-miR-223 | miR-223 | 10.89404 |
| mmu-miR-142-3p | miR-142-3p | 10.78727 |
| mmu-miR-709 | miR-709/1827 | 10.61319 |
| mmu-let-7d | let-7/98/4458/4500 | 10.56519 |
| mmu-let-7g | let-7/98/4458/4500 | 10.42749 |
| mmu-miR-20a | miR-17/17-5p/20ab/20b-5p/93/106ab/427/518a-3p/519d | 10.34566 |
| mmu-miR-106b | miR-17/17-5p/20ab/20b-5p/93/106ab/427/518a-3p/519d | 10.19461 |
| mmu-let-7c | let-7/98/4458/4500 | 10.18402 |
| mmu-miR-23a | miR-23abc/23b-3p | 10.01918 |
| mmu-miR-26a | miR-26ab/1297/4465 | 10.01728 |
| mmu-miR-1224 | miR-1224-5p/1671 | 9.999767 |
| mmu-miR-19a | miR-19ab | 9.977388 |
| mmu-miR-26b | miR-26ab/1297/4465 | 9.955058 |
| mmu-miR-689 | none | 9.930871 |
| mmu-let-7b | let-7/98/4458/4500 | 9.839393 |
| mmu-miR-690 | miR-690 | 9.821926 |
| mmu-let-7i | let-7/98/4458/4500 | 9.709558 |
| mmu-miR-29a | miR-29abcd | 9.62516 |
| mmu-miR-25 | miR-25/32/92abc/363/363-3p/367 | 9.612588 |
| mmu-miR-20b | miR-17/17-5p/20ab/20b-5p/93/106ab/427/518a-3p/519d | 9.552543 |
| mmu-miR-107 | miR-103a/107/107ab | 9.318861 |
| mmu-miR-10a | miR-10abc/10a-5p | 9.257748 |
| mmu-miR-202-3p | miR-202-3p | 9.214806 |
| mmu-miR-23b | miR-23abc/23b-3p | 8.997189 |
| mmu-miR-221 | miR-221/222/222ab/1928 | 8.993728 |
| mmu-miR-494 | miR-494 | 8.964449 |
| mmu-miR-30b | miR-30abcdef/30abe-5p/384-5p | 8.860234 |
| mmu-miR-30c | miR-30abcdef/30abe-5p/384-5p | 8.838458 |
| mmu-miR-24 | miR-24/24ab/24-3p | 8.835341 |
| mmu-miR-15a | miR-15abc/16/16abc/195/322/424/497/1907 | 8.773492 |
| mmu-miR-142-5p | miR-142-5p | 8.773308 |
| mmu-miR-103 | miR-103a/107/107ab | 8.580213 |
| mmu-miR-125b-5p | miR-125a-5p/125b-5p/351/670/4319 | 8.514759 |
| mmu-miR-27a | miR-27abc/27a-3p | 8.389384 |
| mmu-miR-322 | miR-15abc/16/16abc/195/322/424/497/1907 | 8.367191 |
| mmu-miR-16 | miR-15abc/16/16abc/195/322/424/497/1907 | 8.362289 |
| mmu-miR-126-3p | miR-126-3p | 8.361089 |
| mmu-miR-196b | miR-196abc | 8.314435 |
| mmu-miR-30d | miR-30abcdef/30abe-5p/384-5p | 8.229533 |
| mmu-miR-128 | miR-128/128ab | 8.159027 |
| mmu-miR-99a | miR-99ab/100 | 8.111157 |
| mmu-miR-222 | miR-221/222/222ab/1928 | 8.092588 |
| mmu-miR-181a | miR-181abcd/4262 | 8.005014 |
| mmu-miR-148a | miR-148ab-3p/152 | 7.995344 |
| mmu-miR-425 | miR-425/425-5p/489 | 7.962603 |
| mmu-miR-130a | miR-130ac/301ab/301b/301b-3p/454/721/4295/3666 | 7.836963 |
| mmu-miR-30e | miR-30abcdef/30abe-5p/384-5p | 7.819873 |
| mmu-miR-18a | miR-18ab/4735-3p | 7.74359 |
| mmu-miR-140* | none | 7.742659 |
| mmu-miR-93 | miR-17/17-5p/20ab/20b-5p/93/106ab/427/518a-3p/519d | 7.625413 |
| mmu-miR-374 | miR-374ab | 7.600031 |
| mmu-miR-340-5p | miR-340-5p | 7.537324 |
| mmu-miR-29b | miR-29abcd | 7.504763 |
| mmu-miR-181c | miR-181abcd/4262 | 7.498703 |
| mmu-miR-212 | none | 7.496817 |
| mmu-miR-130b | miR-130ac/301ab/301b/301b-3p/454/721/4295/3666 | 7.410023 |
| mmu-miR-574-5p | miR-574-5p | 7.37567 |
| mmu-miR-181b | miR-181abcd/4262 | 7.309724 |
| mmu-miR-301a | miR-130ac/301ab/301b/301b-3p/454/721/4295/3666 | 7.303752 |
| mmu-miR-29c | miR-29abcd | 7.265734 |
| mmu-miR-671-5p | miR-671-5p | 7.225901 |
| mmu-let-7e | let-7/98/4458/4500 | 7.223696 |
| mmu-miR-697 | miR-697 | 7.198383 |
| mmu-miR-342-3p | miR-342-3p | 7.09636 |
| mmu-miR-17* | none | 7.090929 |
| mmu-miR-98 | let-7/98/4458/4500 | 7.089791 |
| mmu-miR-155 | miR-155 | 6.962562 |
| mmu-miR-350 | miR-350 | 6.866321 |
| mmu-miR-27b | miR-27abc/27a-3p | 6.849862 |
| mmu-miR-17 | miR-17/17-5p/20ab/20b-5p/93/106ab/427/518a-3p/519d | 6.849556 |
| mmu-miR-150 | miR-150/5127 | 6.843909 |
| mmu-miR-450a-5p | none | 6.816891 |
| mmu-miR-195 | miR-15abc/16/16abc/195/322/424/497/1907 | 6.780849 |
| mmu-miR-101a | miR-101/101ab | 6.753674 |
| mmu-miR-487b | miR-487b | 6.740535 |
| mmu-miR-106a | miR-17/17-5p/20ab/20b-5p/93/106ab/427/518a-3p/519d | 6.737892 |
| mmu-miR-652 | miR-652 | 6.732245 |
| mmu-miR-181d | miR-181abcd/4262 | 6.716534 |
| mmu-miR-203 | miR-203 | 6.700049 |
| mmu-miR-451 | miR-451 | 6.68382 |
| mmu-miR-146b | miR-146ac/146b-5p | 6.683588 |
| mmu-miR-101b | miR-101/101ab | 6.683105 |
| mmu-miR-370 | miR-370 | 6.641437 |
| mmu-miR-712 | miR-712 | 6.631147 |
| mmu-miR-345-5p | miR-345-5p.m | 6.60606 |
| mmu-miR-188-5p | miR-188-5p | 6.567046 |
| mmu-miR-34a | miR-34ac/34bc-5p/449abc/449c-5p | 6.540818 |
| mmu-miR-324-3p | miR-324-3p | 6.52831 |
| mmu-miR-721 | miR-130ac/301ab/301b/301b-3p/454/721/4295/3666 | 6.514925 |
| mmu-miR-680 | miR-680 | 6.485956 |
| mmu-miR-801 | none | 6.468586 |
| mmu-miR-140 | miR-140/140-5p/876-3p/1244 | 6.466564 |
| mmu-miR-30a | miR-30abcdef/30abe-5p/384-5p | 6.45969 |
| mmu-miR-423-5p | miR-423a/423-5p/3184/3573-5p | 6.427837 |
| mmu-miR-878-3p | miR-878-3p | 6.413092 |
| mmu-miR-362-3p | miR-329/329ab/362-3p | 6.406925 |
| mmu-miR-714 | miR-714 | 6.402224 |
| mmu-miR-219 | none | 6.388146 |
| mmu-miR-467b | miR-467b | 6.31636 |
| mmu-miR-378 | miR-378/422a/378bcdefhi | 6.315916 |
| mmu-miR-361 | miR-361-5p | 6.292908 |
| mmu-miR-320 | miR-320abcd/4429 | 6.264433 |

Table S1d: miRs expressed over 100 copies per cell in the CMP Population

| **miR** | **Family** | **Normalized Array Intensity** |
| --- | --- | --- |
| mmu-miR-142-3p | miR-142-3p | 12.60349 |
| mmu-miR-720 | miR-720.m | 12.46316 |
| mmu-miR-19b | miR-19ab | 12.01886 |
| mmu-miR-223 | miR-223 | 11.79071 |
| mmu-miR-21 | miR-21/590-5p | 11.44021 |
| mmu-miR-92a | miR-25/32/92abc/363/363-3p/367 | 11.23672 |
| mmu-miR-20a | miR-17/17-5p/20ab/20b-5p/93/106ab/427/518a-3p/519d | 11.12965 |
| mmu-let-7f | let-7/98/4458/4500 | 10.84466 |
| mmu-miR-15b | miR-15abc/16/16abc/195/322/424/497/1907 | 10.64145 |
| mmu-miR-16 | miR-15abc/16/16abc/195/322/424/497/1907 | 10.61857 |
| mmu-let-7i | let-7/98/4458/4500 | 10.42563 |
| mmu-miR-20b | miR-17/17-5p/20ab/20b-5p/93/106ab/427/518a-3p/519d | 10.42312 |
| mmu-miR-709 | miR-709/1827 | 10.2852 |
| mmu-let-7a | let-7/98/4458/4500 | 10.27544 |
| mmu-miR-19a | miR-19ab | 10.21199 |
| mmu-miR-106b | miR-17/17-5p/20ab/20b-5p/93/106ab/427/518a-3p/519d | 10.11225 |
| mmu-miR-1224 | miR-1224-5p/1671 | 9.983205 |
| mmu-miR-15a | miR-15abc/16/16abc/195/322/424/497/1907 | 9.953745 |
| mmu-let-7g | let-7/98/4458/4500 | 9.904879 |
| mmu-miR-26a | miR-26ab/1297/4465 | 9.881613 |
| mmu-miR-690 | miR-690 | 9.74871 |
| mmu-miR-29a | miR-29abcd | 9.731035 |
| mmu-miR-142-5p | miR-142-5p | 9.666197 |
| mmu-let-7d | let-7/98/4458/4500 | 9.630415 |
| mmu-miR-25 | miR-25/32/92abc/363/363-3p/367 | 9.629379 |
| mmu-miR-23a | miR-23abc/23b-3p | 9.621658 |
| mmu-miR-494 | miR-494 | 9.55295 |
| mmu-miR-29b | miR-29abcd | 9.444184 |
| mmu-miR-26b | miR-26ab/1297/4465 | 9.143863 |
| mmu-miR-24 | miR-24/24ab/24-3p | 9.143237 |
| mmu-miR-27a | miR-27abc/27a-3p | 9.058417 |
| mmu-miR-107 | miR-103a/107/107ab | 9.043319 |
| mmu-miR-93 | miR-17/17-5p/20ab/20b-5p/93/106ab/427/518a-3p/519d | 9.007948 |
| mmu-let-7c | let-7/98/4458/4500 | 8.924406 |
| mmu-miR-130a | miR-130ac/301ab/301b/301b-3p/454/721/4295/3666 | 8.911016 |
| mmu-miR-221 | miR-221/222/222ab/1928 | 8.773715 |
| mmu-miR-23b | miR-23abc/23b-3p | 8.743892 |
| mmu-miR-18a | miR-18ab/4735-3p | 8.659672 |
| mmu-miR-30b | miR-30abcdef/30abe-5p/384-5p | 8.593093 |
| mmu-let-7b | let-7/98/4458/4500 | 8.529055 |
| mmu-miR-30c | miR-30abcdef/30abe-5p/384-5p | 8.429485 |
| mmu-miR-30e | miR-30abcdef/30abe-5p/384-5p | 8.417092 |
| mmu-miR-322 | miR-15abc/16/16abc/195/322/424/497/1907 | 8.348446 |
| mmu-miR-181a | miR-181abcd/4262 | 8.34126 |
| mmu-miR-27b | miR-27abc/27a-3p | 8.335461 |
| mmu-miR-103 | miR-103a/107/107ab | 8.301043 |
| mmu-miR-17 | miR-17/17-5p/20ab/20b-5p/93/106ab/427/518a-3p/519d | 8.176979 |
| mmu-miR-130b | miR-130ac/301ab/301b/301b-3p/454/721/4295/3666 | 8.154175 |
| mmu-miR-425 | miR-425/425-5p/489 | 8.142039 |
| mmu-miR-148a | miR-148ab-3p/152 | 8.122168 |
| mmu-miR-106a | miR-17/17-5p/20ab/20b-5p/93/106ab/427/518a-3p/519d | 8.103419 |
| mmu-miR-181c | miR-181abcd/4262 | 7.991858 |
| mmu-miR-30d | miR-30abcdef/30abe-5p/384-5p | 7.971504 |
| mmu-miR-181b | miR-181abcd/4262 | 7.858246 |
| mmu-miR-652 | miR-652 | 7.776294 |
| mmu-miR-17* | None | 7.765113 |
| mmu-miR-140* | None | 7.651824 |
| mmu-miR-29c | miR-29abcd | 7.615221 |
| mmu-miR-689 | None | 7.522706 |
| mmu-miR-126-3p | miR-126-3p | 7.486166 |
| mmu-miR-340-5p | miR-340-5p | 7.468703 |
| mmu-miR-301a | miR-130ac/301ab/301b/301b-3p/454/721/4295/3666 | 7.45598 |
| mmu-miR-374 | miR-374ab | 7.455388 |
| mmu-miR-222 | miR-221/222/222ab/1928 | 7.397366 |
| mmu-miR-10a | miR-10abc/10a-5p | 7.395579 |
| mmu-miR-125b-5p | miR-125a-5p/125b-5p/351/670/4319 | 7.392001 |
| mmu-miR-181d | miR-181abcd/4262 | 7.298083 |
| mmu-miR-101b | miR-101/101ab | 7.250974 |
| mmu-miR-714 | miR-714 | 7.246571 |
| mmu-miR-196b | miR-196abc | 7.219618 |
| mmu-miR-99a | miR-99ab/100 | 7.190727 |
| mmu-miR-140 | miR-140/140-5p/876-3p/1244 | 7.164617 |
| mmu-miR-202-3p | miR-202-3p | 7.133434 |
| mmu-miR-574-5p | miR-574-5p | 7.109074 |
| mmu-miR-451 | miR-451 | 7.106766 |
| mmu-miR-101a | miR-101/101ab | 7.055572 |
| mmu-miR-350 | miR-350 | 7.011693 |
| mmu-miR-98 | let-7/98/4458/4500 | 6.994216 |
| mmu-miR-450a-5p | none | 6.942915 |
| mmu-miR-128 | miR-128/128ab | 6.862692 |
| mmu-miR-378 | miR-378/422a/378bcdefhi | 6.796127 |
| mmu-miR-34a | miR-34ac/34bc-5p/449abc/449c-5p | 6.791057 |
| mmu-miR-671-5p | miR-671-5p | 6.763712 |
| mmu-miR-155 | miR-155 | 6.753121 |
| mmu-miR-212 | none | 6.708094 |
| mmu-miR-721 | miR-130ac/301ab/301b/301b-3p/454/721/4295/3666 | 6.666562 |
| mmu-miR-345-5p | miR-345-5p.m | 6.613853 |
| mmu-miR-30a | miR-30abcdef/30abe-5p/384-5p | 6.499266 |
| mmu-miR-370 | miR-370 | 6.495082 |
| mmu-miR-219 | none | 6.462039 |
| mmu-miR-324-5p | miR-324-5p | 6.397123 |
| mmu-miR-483 | miR-483-5p | 6.383135 |
| mmu-miR-7a | miR-7/7ab | 6.374948 |
| mmu-miR-146b | miR-146ac/146b-5p | 6.357243 |
| mmu-miR-712 | miR-712 | 6.357016 |
| mmu-miR-361 | miR-361-5p | 6.350267 |
| mmu-miR-680 | miR-680 | 6.337251 |
| mmu-miR-185 | miR-185/882/3473/4306/4644 | 6.311675 |
| mmu-miR-135a* | 135a* | 6.307268 |
| mmu-miR-423-5p | miR-423a/423-5p/3184/3573-5p | 6.287538 |
| mmu-miR-801 | none | 6.285143 |
| mmu-miR-342-3p | miR-342-3p | 6.271771 |

Table S1e: miRs expressed over 100 copies per cell in the GMP Population

| **miR** | **Family** | **Normalized Array Intensity** |
| --- | --- | --- |
| mmu-miR-223 | miR-223 | 13.19588 |
| mmu-miR-142-3p | miR-142-3p | 12.49611 |
| mmu-miR-720 | miR-720.m | 12.35311 |
| mmu-miR-19b | miR-19ab | 11.68868 |
| mmu-miR-20a | miR-17/17-5p/20ab/20b-5p/93/106ab/427/518a-3p/519d | 11.2803 |
| mmu-miR-92a | miR-25/32/92abc/363/363-3p/367 | 11.0389 |
| mmu-let-7f | let-7/98/4458/4500 | 10.93622 |
| mmu-miR-16 | miR-15abc/16/16abc/195/322/424/497/1907 | 10.86496 |
| mmu-miR-21 | miR-21/590-5p | 10.77583 |
| mmu-miR-15b | miR-15abc/16/16abc/195/322/424/497/1907 | 10.6818 |
| mmu-miR-20b | miR-17/17-5p/20ab/20b-5p/93/106ab/427/518a-3p/519d | 10.63147 |
| mmu-miR-494 | miR-494 | 10.56106 |
| mmu-let-7a | let-7/98/4458/4500 | 10.39098 |
| mmu-miR-19a | miR-19ab | 10.3708 |
| mmu-let-7i | let-7/98/4458/4500 | 10.18677 |
| mmu-miR-709 | miR-709/1827 | 10.139 |
| mmu-miR-1224 | miR-1224-5p/1671 | 10.0417 |
| mmu-let-7g | let-7/98/4458/4500 | 10.01445 |
| mmu-miR-142-5p | miR-142-5p | 9.879317 |
| mmu-miR-15a | miR-15abc/16/16abc/195/322/424/497/1907 | 9.86392 |
| mmu-miR-106b | miR-17/17-5p/20ab/20b-5p/93/106ab/427/518a-3p/519d | 9.856128 |
| mmu-miR-690 | miR-690 | 9.767931 |
| mmu-miR-23a | miR-23abc/23b-3p | 9.738688 |
| mmu-let-7d | let-7/98/4458/4500 | 9.563196 |
| mmu-miR-25 | miR-25/32/92abc/363/363-3p/367 | 9.426487 |
| mmu-miR-29a | miR-29abcd | 9.422998 |
| mmu-miR-148a | miR-148ab-3p/152 | 9.419093 |
| mmu-miR-26a | miR-26ab/1297/4465 | 9.394405 |
| mmu-miR-27a | miR-27abc/27a-3p | 9.358446 |
| mmu-miR-24 | miR-24/24ab/24-3p | 9.267609 |
| mmu-miR-93 | miR-17/17-5p/20ab/20b-5p/93/106ab/427/518a-3p/519d | 9.233209 |
| mmu-miR-29b | miR-29abcd | 9.213483 |
| mmu-miR-18a | miR-18ab/4735-3p | 9.195494 |
| mmu-miR-26b | miR-26ab/1297/4465 | 8.931026 |
| mmu-miR-107 | miR-103a/107/107ab | 8.844223 |
| mmu-let-7c | let-7/98/4458/4500 | 8.743184 |
| mmu-miR-221 | miR-221/222/222ab/1928 | 8.69892 |
| mmu-miR-23b | miR-23abc/23b-3p | 8.671056 |
| mmu-let-7b | let-7/98/4458/4500 | 8.51115 |
| mmu-miR-30b | miR-30abcdef/30abe-5p/384-5p | 8.488425 |
| mmu-miR-103 | miR-103a/107/107ab | 8.458578 |
| mmu-miR-30e | miR-30abcdef/30abe-5p/384-5p | 8.458229 |
| mmu-miR-17 | miR-17/17-5p/20ab/20b-5p/93/106ab/427/518a-3p/519d | 8.430862 |
| mmu-miR-27b | miR-27abc/27a-3p | 8.413707 |
| mmu-miR-106a | miR-17/17-5p/20ab/20b-5p/93/106ab/427/518a-3p/519d | 8.363575 |
| mmu-miR-130b | miR-130ac/301ab/301b/301b-3p/454/721/4295/3666 | 8.281694 |
| mmu-miR-425 | miR-425/425-5p/489 | 8.156373 |
| mmu-miR-30c | miR-30abcdef/30abe-5p/384-5p | 8.122032 |
| mmu-miR-30d | miR-30abcdef/30abe-5p/384-5p | 8.006115 |
| mmu-miR-652 | miR-652 | 7.967845 |
| mmu-miR-340-5p | miR-340-5p | 7.833294 |
| mmu-miR-202-3p | miR-202-3p | 7.772091 |
| mmu-miR-140* | None | 7.768561 |
| mmu-miR-130a | miR-130ac/301ab/301b/301b-3p/454/721/4295/3666 | 7.692132 |
| mmu-miR-181b | miR-181abcd/4262 | 7.683084 |
| mmu-miR-17* | None | 7.681205 |
| mmu-miR-181a | miR-181abcd/4262 | 7.636567 |
| mmu-miR-301a | miR-130ac/301ab/301b/301b-3p/454/721/4295/3666 | 7.598469 |
| mmu-miR-322 | miR-15abc/16/16abc/195/322/424/497/1907 | 7.552873 |
| mmu-miR-689 | None | 7.546116 |
| mmu-miR-29c | miR-29abcd | 7.502452 |
| mmu-miR-181c | miR-181abcd/4262 | 7.494142 |
| mmu-miR-374 | miR-374ab | 7.437494 |
| mmu-miR-101b | miR-101/101ab | 7.419377 |
| mmu-miR-146b | miR-146ac/146b-5p | 7.4161 |
| mmu-miR-98 | let-7/98/4458/4500 | 7.385806 |
| mmu-miR-140 | miR-140/140-5p/876-3p/1244 | 7.331091 |
| mmu-miR-222 | miR-221/222/222ab/1928 | 7.294986 |
| mmu-miR-10a | miR-10abc/10a-5p | 7.249105 |
| mmu-miR-378 | miR-378/422a/378bcdefhi | 7.227443 |
| mmu-miR-212 | none | 7.198516 |
| mmu-miR-181d | miR-181abcd/4262 | 7.146083 |
| mmu-miR-574-5p | miR-574-5p | 7.124609 |
| mmu-miR-721 | miR-130ac/301ab/301b/301b-3p/454/721/4295/3666 | 7.086328 |
| mmu-miR-338-3p | miR-338/338-3p | 7.057242 |
| mmu-miR-350 | miR-350 | 7.034221 |
| mmu-miR-101a | miR-101/101ab | 6.939811 |
| mmu-miR-714 | miR-714 | 6.864985 |
| mmu-miR-450a-5p | None | 6.824026 |
| mmu-miR-801 | None | 6.800348 |
| mmu-miR-370 | miR-370 | 6.791547 |
| mmu-miR-7a | miR-7/7ab | 6.735471 |
| mmu-miR-22 | miR-22/22-3p | 6.710073 |
| mmu-miR-345-5p | miR-345-5p.m | 6.705728 |
| mmu-miR-125b-5p | miR-125a-5p/125b-5p/351/670/4319 | 6.673525 |
| mmu-miR-30a | miR-30abcdef/30abe-5p/384-5p | 6.58144 |
| mmu-miR-219 | none | 6.534452 |
| mmu-miR-99a | miR-99ab/100 | 6.527184 |
| mmu-miR-712 | miR-712 | 6.512815 |
| mmu-miR-149 | miR-149 | 6.512352 |
| mmu-miR-126-3p | miR-126-3p | 6.508956 |
| mmu-miR-423-5p | miR-423a/423-5p/3184/3573-5p | 6.503888 |
| mmu-miR-155 | miR-155 | 6.486457 |
| mmu-miR-361 | miR-361-5p | 6.476696 |
| mmu-miR-148b | miR-148ab-3p/152 | 6.472748 |
| mmu-miR-483 | miR-483-5p | 6.470834 |
| mmu-miR-680 | miR-680 | 6.411393 |
| mmu-miR-34a | miR-34ac/34bc-5p/449abc/449c-5p | 6.410691 |
| mmu-miR-196b | miR-196abc | 6.398453 |
| mmu-miR-188-5p | miR-188-5p | 6.39589 |
| mmu-miR-324-5p | miR-324-5p | 6.381523 |
| mmu-miR-185 | miR-185/882/3473/4306/4644 | 6.372884 |
| mmu-miR-342-3p | miR-342-3p | 6.365939 |
| mmu-miR-340-3p | miR-340-3p | 6.280365 |

Table S1f: miRs expressed over 100 copies per cell in the MEP Population

| **miR** | **Family** | **Normalized Array Intensity** |
| --- | --- | --- |
| mmu-miR-1224 | miR-1224-5p/1671 | 12.3925 |
| mmu-miR-142-3p | miR-142-3p | 12.15215 |
| mmu-miR-720 | miR-720.m | 11.85386 |
| mmu-miR-494 | miR-494 | 11.79192 |
| mmu-miR-451 | miR-451 | 11.7403 |
| mmu-miR-19b | miR-19ab | 11.44151 |
| mmu-miR-20a | miR-17/17-5p/20ab/20b-5p/93/106ab/427/518a-3p/519d | 11.08428 |
| mmu-miR-709 | miR-709/1827 | 11.0763 |
| mmu-miR-92a | miR-25/32/92abc/363/363-3p/367 | 10.84681 |
| mmu-miR-21 | miR-21/590-5p | 10.6854 |
| mmu-miR-19a | miR-19ab | 10.5713 |
| mmu-let-7f | let-7/98/4458/4500 | 10.51784 |
| mmu-miR-20b | miR-17/17-5p/20ab/20b-5p/93/106ab/427/518a-3p/519d | 10.48515 |
| mmu-miR-15b | miR-15abc/16/16abc/195/322/424/497/1907 | 10.40178 |
| mmu-miR-16 | miR-15abc/16/16abc/195/322/424/497/1907 | 10.3495 |
| mmu-miR-690 | miR-690 | 10.2428 |
| mmu-let-7a | let-7/98/4458/4500 | 10.00302 |
| mmu-miR-142-5p | miR-142-5p | 9.872218 |
| mmu-let-7i | let-7/98/4458/4500 | 9.808269 |
| mmu-miR-106b | miR-17/17-5p/20ab/20b-5p/93/106ab/427/518a-3p/519d | 9.71081 |
| mmu-let-7g | let-7/98/4458/4500 | 9.685983 |
| mmu-miR-574-5p | miR-574-5p | 9.593912 |
| mmu-miR-25 | miR-25/32/92abc/363/363-3p/367 | 9.499778 |
| mmu-miR-15a | miR-15abc/16/16abc/195/322/424/497/1907 | 9.461259 |
| mmu-miR-18a | miR-18ab/4735-3p | 9.424792 |
| mmu-let-7d | let-7/98/4458/4500 | 9.407122 |
| mmu-miR-714 | miR-714 | 9.334223 |
| mmu-miR-144 | miR-144 | 9.302722 |
| mmu-miR-689 | none | 9.262249 |
| mmu-miR-223 | miR-223 | 9.25436 |
| mmu-miR-370 | miR-370 | 9.19413 |
| mmu-miR-721 | miR-130ac/301ab/301b/301b-3p/454/721/4295/3666 | 9.040483 |
| mmu-miR-93 | miR-17/17-5p/20ab/20b-5p/93/106ab/427/518a-3p/519d | 8.989017 |
| mmu-miR-29a | miR-29abcd | 8.876002 |
| mmu-miR-107 | miR-103a/107/107ab | 8.756711 |
| mmu-miR-26b | miR-26ab/1297/4465 | 8.682368 |
| mmu-miR-26a | miR-26ab/1297/4465 | 8.588283 |
| mmu-let-7c | let-7/98/4458/4500 | 8.57933 |
| mmu-miR-29b | miR-29abcd | 8.467018 |
| mmu-miR-202-3p | miR-202-3p | 8.421167 |
| mmu-let-7b | let-7/98/4458/4500 | 8.335767 |
| mmu-miR-680 | miR-680 | 8.333572 |
| mmu-miR-17 | miR-17/17-5p/20ab/20b-5p/93/106ab/427/518a-3p/519d | 8.32202 |
| mmu-miR-671-5p | miR-671-5p | 8.291501 |
| mmu-miR-106a | miR-17/17-5p/20ab/20b-5p/93/106ab/427/518a-3p/519d | 8.216643 |
| mmu-miR-103 | miR-103a/107/107ab | 8.121495 |
| mmu-miR-30e | miR-30abcdef/30abe-5p/384-5p | 8.097961 |
| mmu-miR-483 | miR-483-5p | 8.097602 |
| mmu-miR-30b | miR-30abcdef/30abe-5p/384-5p | 8.076351 |
| mmu-miR-712 | miR-712 | 8.066792 |
| mmu-miR-188-5p | miR-188-5p | 8.01991 |
| mmu-miR-486 | miR-486-5p/3107 | 7.955187 |
| mmu-miR-30c | miR-30abcdef/30abe-5p/384-5p | 7.844566 |
| mmu-miR-148a | miR-148ab-3p/152 | 7.616509 |
| mmu-miR-24 | miR-24/24ab/24-3p | 7.594753 |
| mmu-miR-425 | miR-425/425-5p/489 | 7.587272 |
| mmu-miR-130b | miR-130ac/301ab/301b/301b-3p/454/721/4295/3666 | 7.520177 |
| mmu-miR-30d | miR-30abcdef/30abe-5p/384-5p | 7.51152 |
| mmu-miR-705 | miR-705/2897 | 7.505409 |
| mmu-miR-17* | none | 7.474984 |
| mmu-miR-378 | miR-378/422a/378bcdefhi | 7.453054 |
| mmu-miR-23b | miR-23abc/23b-3p | 7.446776 |
| mmu-miR-7a | miR-7/7ab | 7.434231 |
| mmu-miR-374 | miR-374ab | 7.347337 |
| mmu-miR-101b | miR-101/101ab | 7.323402 |
| mmu-miR-23a | miR-23abc/23b-3p | 7.319181 |
| mmu-miR-135a* | 135a* | 7.261581 |
| mmu-miR-29c | miR-29abcd | 7.238301 |
| mmu-miR-652 | miR-652 | 7.211792 |
| mmu-miR-712* | None | 7.206664 |
| mmu-miR-801 | none | 7.143126 |
| mmu-miR-185 | miR-185/882/3473/4306/4644 | 7.068629 |
| mmu-miR-762 | miR-762/4492/4498 | 6.987475 |
| mmu-miR-341 | miR-341 | 6.983403 |
| mmu-miR-212 | None | 6.950258 |
| mmu-miR-27b | miR-27abc/27a-3p | 6.888835 |
| mmu-miR-322 | miR-15abc/16/16abc/195/322/424/497/1907 | 6.888663 |
| mmu-miR-669c | miR-669c | 6.88586 |
| mmu-miR-140* | none | 6.884541 |
| mmu-miR-34a | miR-34ac/34bc-5p/449abc/449c-5p | 6.859491 |
| mmu-miR-98 | let-7/98/4458/4500 | 6.824205 |
| mmu-miR-221 | miR-221/222/222ab/1928 | 6.804074 |
| mmu-miR-706 | miR-706 | 6.787275 |
| mmu-miR-101a | miR-101/101ab | 6.779884 |
| mmu-miR-139-3p | miR-139-3p.dmr | 6.748802 |
| mmu-miR-30a | miR-30abcdef/30abe-5p/384-5p | 6.718219 |
| mmu-miR-27a | miR-27abc/27a-3p | 6.667463 |
| mmu-miR-301a | miR-130ac/301ab/301b/301b-3p/454/721/4295/3666 | 6.664654 |
| mmu-miR-125a-3p | miR-125a-3p/1554 | 6.617689 |
| mmu-miR-181c | miR-181abcd/4262 | 6.6149 |
| mmu-miR-350 | miR-350 | 6.568378 |
| mmu-miR-340-5p | miR-340-5p | 6.479136 |
| mmu-miR-181a | miR-181abcd/4262 | 6.47755 |
| mmu-miR-320 | miR-320abcd/4429 | 6.459106 |
| mmu-miR-219 | None | 6.452874 |
| mmu-miR-140 | miR-140/140-5p/876-3p/1244 | 6.422658 |
| mmu-miR-452 | none | 6.41352 |
| mmu-miR-290-5p | miR-290-5p/292-5p/371-5p/293 | 6.401817 |
| mmu-miR-345-5p | miR-345-5p.m | 6.398211 |
| mmu-miR-466f-3p | miR-466f-3p | 6.386141 |
| mmu-miR-126-3p | miR-126-3p | 6.380358 |
| mmu-miR-181d | miR-181abcd/4262 | 6.344953 |
| mmu-miR-423-5p | miR-423a/423-5p/3184/3573-5p | 6.292496 |
| mmu-miR-805 | none | 6.28964 |
| mmu-miR-466c-5p | miR-466bco-5p | 6.282918 |
| mmu-miR-128 | miR-128/128ab | 6.276784 |
| mmu-miR-134 | miR-134/3118 | 6.25592 |
