# Supplementary material for: Correlated miR-mRNA Expression Signatures of Mouse Hematopoietic Stem and Progenitor Cell Subsets Predict “Stemness” and “Myeloid” Interaction Networks
Source: PLoS One. 2014 Apr 18;9(4):e94852. doi: 10.1371/journal.pone.0094852 (PMC3991639; doi:10.1371/journal.pone.0094852)
Supplement: Table S2 — miR family expression in HSPC subsets. miRs are grouped by family, and HSPC populations expressing each family over 100 copies per cell are listed. ALL indicates that the miR family is represented over the threshold in all HSPC populations examined (LT-HSC, ST-HSC, MPP, CMP, GMP and MEP). If the miR is not a member of a known family, the family listed refers to the individual microRNA itself (mmu-miR-xx) (DOCX) [file pone.0094852.s002.docx]

**Table S2: miR family expression in HSPC subsets**

| **miR Family** | **Populations expressing over 100 copies/cell** |
| --- | --- |
| mmu-miR-689 | ALL |
| mmu-miR-801 | ALL |
| mmu-miR-212 | ALL |
| mmu-miR-140* | ALL |
| let-7/98/4458/4500 | ALL |
| miR-101/101ab | ALL |
| miR-103a/107/107ab | ALL |
| miR-1224-5p/1671 | ALL |
| miR-126-3p | ALL |
| miR-130ac/301ab/301b/301b-3p/454/721/4295/3666 | ALL |
| miR-140/140-5p/876-3p/1244 | ALL |
| miR-142-3p | ALL |
| miR-142-5p | ALL |
| miR-148ab-3p/152 | ALL |
| miR-15abc/16/16abc/195/322/424/497/1907 | ALL |
| miR-17/17-5p/20ab/20b-5p/93/106ab/427/518a-3p/519d | ALL |
| miR-181abcd/4262 | ALL |
| miR-18ab/4735-3p | ALL |
| miR-19ab | ALL |
| miR-202-3p | ALL |
| miR-21/590-5p | ALL |
| miR-221/222/222ab/1928 | ALL |
| miR-223 | ALL |
| miR-23abc/23b-3p | ALL |
| miR-24/24ab/24-3p | ALL |
| miR-25/32/92abc/363/363-3p/367 | ALL |
| miR-26ab/1297/4465 | ALL |
| miR-27abc/27a-3p | ALL |
| miR-29abcd | ALL |
| miR-30abcdef/30abe-5p/384-5p | ALL |
| miR-345-5p.m | ALL |
| miR-34ac/34bc-5p/449abc/449c-5p | ALL |
| miR-370 | ALL |
| miR-374ab | ALL |
| miR-378/422a/378bcdefhi | ALL |
| miR-425/425-5p/489 | ALL |
| miR-494 | ALL |
| miR-574-5p | ALL |
| miR-652 | ALL |
| miR-680 | ALL |
| miR-690 | ALL |
| miR-709/1827 | ALL |
| miR-712 | ALL |
| miR-714 | ALL |
| miR-720.m | ALL |
| mmu-miR-17* | ALL |
| miR-188-5p | LT-HSC, ST-HSC, MPP, GMP, MEP |
| miR-451 | LT-HSC, ST-HSC, MPP, CMP, MEP |
| miR-671-5p | LT-HSC, ST-HSC, MPP, CMP, MEP |
| miR-340-5p | ST-HSC, MPP, CMP, GMP, MEP |
| miR-350 | ST-HSC, MPP, CMP, GMP, MEP |
| miR-10abc/10a-5p | LT-HSC, ST-HSC, MPP, CMP, GMP |
| miR-125a-5p/125b-5p/351/670/4319 | LT-HSC, ST-HSC, MPP, CMP, GMP |
| miR-146ac/146b-5p | LT-HSC, ST-HSC, MPP, CMP, GMP |
| miR-155 | LT-HSC, ST-HSC, MPP, CMP, GMP |
| miR-196abc | LT-HSC, ST-HSC, MPP, CMP, GMP |
| miR-99ab/100 | LT-HSC, ST-HSC, MPP, CMP, GMP |
| miR-483-5p | LT-HSC, ST-HSC, MPP, CMP, GMP |
| miR-324-5p | CMP, GMP |
| miR-185/882/3473/4306/4644 | CMP, GMP, MEP |
| miR-7/7ab | CMP, GMP, MEP |
| miR-149 | GMP |
| miR-338/338-3p | GMP |
| miR-340-3p | GMP |
| 135a* | LT-HSC, CMP, MEP |
| miR-22/22-3p | LT-HSC, GMP |
| miR-144 | LT-HSC, MEP |
| miR-290-5p/292-5p/371-5p/293 | LT-HSC, MEP |
| miR-341 | LT-HSC, MEP |
| miR-486-5p/3107 | LT-HSC, MEP |
| miR-669c | LT-HSC, MEP |
| miR-706 | LT-HSC, MEP |
| miR-762/4492/4498 | LT-HSC, MEP |
| miR-150/5127 | LT-HSC, MPP |
| miR-125a-3p/1554 | LT-HSC, ST-HSC, MEP |
| miR-139-3p.dmr | LT-HSC, ST-HSC, MEP |
| miR-705/2897 | LT-HSC, ST-HSC, MEP |
| miR-320abcd/4429 | LT-HSC, ST-HSC, MPP, MEP |
| miR-697 | LT-HSC, ST-HSC, MPP |
| mmu-miR-877* | LT-HSC |
| mmu-miR-712* | MEP |
| mmu-miR-219 | MEP |
| mmu-miR-452 | MEP |
| mmu-miR-805 | MEP |
| miR-134/3118 | MEP |
| miR-466bco-5p | MEP |
| miR-466f-3p | MEP |
| miR-342-3p | MPP, CMP, GMP |
| miR-361-5p | MPP, CMP, GMP |
| miR-423a/423-5p/3184/3573-5p | MPP, CMP, GMP, MEP |
| miR-329/329ab/362-3p | MPP |
| miR-467b | MPP |
| miR-878-3p | MPP |
| miR-324-3p | ST-HSC, MPP |
| mmu-miR-450a-5p | ST-HSC, MPP, CMP, GMP |
| miR-128/128ab | ST-HSC, MPP, CMP, MEP |
| miR-203 | ST-HSC, MPP |
| miR-487b | ST-HSC, MPP |
