# Supplementary material for: Correlated miR-mRNA Expression Signatures of Mouse Hematopoietic Stem and Progenitor Cell Subsets Predict “Stemness” and “Myeloid” Interaction Networks
Source: PLoS One. 2014 Apr 18;9(4):e94852. doi: 10.1371/journal.pone.0094852 (PMC3991639; doi:10.1371/journal.pone.0094852)
Supplement: Table S4 — Predicted targets of GMP miRs. 242 GMP targets predicted by TargetScan and/or MiRanda to have binding sites for at least one “GMP miR”. Additionally, each target shows a statistically significant inverse pattern of expression with its targeting miR, across our 6 HSPC populations (p<0.05). (DOCX) [file pone.0094852.s004.docx]

**Table S4: Predicted targets of “GMP miRs”**

| **GMP Targets** | | | |
| --- | --- | --- | --- |
| \| Tmem120b \| \| --- \| \| Vamp5 \| \| Snn \| \| Csf1 \| \| Itga9 \| \| 5830405N20Rik \| \| AW555464 \| \| Slco3a1 \| \| Prnp \| \| Klf4 \| \| Sash1 \| \| Cd72 \| \| Pea15a \| \| Notch1 \| \| Btaf1 \| \| Pde1b \| \| Dbp \| \| Slc11a2 \| \| 1300018I17Rik \| \| Mllt6 \| \| Slc30a4 \| \| Arhgef17 \| \| Trps1 \| \| Met \| \| Zfhx3 \| \| Ppp1r9a \| \| Maf \| \| Hlf \| \| Arhgef12 \| \| Mboat2 \| \| Nfat5 \| \| Tfrc \| \| Bach2 \| \| Limd2 \| \| Tbc1d10c \| \| Il6st \| \| Slc2a1 \| \| Gstm2 \| \| Nras \| \| Abcb7 \| \| C530008M17Rik \| \| Zfp516 \| \| Cpm \| \| Spire1 \| \| Epas1 \| \| Ociad2 \| \| Cerk \| \| Ypel1 \| \| Tspan6 \| \| Tbkbp1 \| \| Rgl1 \| \| Tacc2 \| \| Ccnk \| \| Dnmt3b \| \| Reln \| \| Mast4 \| \| Tsc22d1 \| | \| Vdr \| \| \| --- \| --- \| \| Rxrg \| \| \| Lass6 \| \| \| B4galt4 \| \| \| Socs3 \| \| \| Stxbp6 \| \| \| Gusb \| \| \| Tspan33 \| \| \| Spsb4 \| \| \| Fbxo21 \| \| \| Dgka \| \| \| Pdgfrb \| \| \| Micall1 \| \| \| Spnb1 \| \| \| Ppap2a \| \| \| Hsd17b11 \| \| \| Crim1 \| \| Bace1 \| \| Fbxo10 \| \| Tmem121 \| \| Slc22a23 \| \| Atp8b4 \| \| Nr4a1 \| \| Nsg1 \| \| Dclk2 \| \| Gpr56 \| \| Pou2af1 \| \| Sorbs1 \| \| Adcy6 \| \| Rassf4 \| \| Chd3 \| \| Rab37 \| \| 1500009L16Rik \| \| Csrp2 \| \| 9830001H06Rik \| \| Narf \| \| Junb \| \| Cd2ap \| \| Sept8 \| \| Rgs2 \| \| Mycn \| \| Col5a1 \| \| Epb4.1l4b \| \| Paqr4 \| \| Srpk2 \| \| Aldh5a1 \| \| Pgam1 \| \| Nfatc1 \| \| Shank3 \| \| Trp53inp1 \| \| Spnb2 \| \| Abcg4 \| \| Glud1 \| \| Nfia \| \| Arid5b \| \| Pdlim2 \| \| Plekha2 \| \| Arhgef6 \| \| Armcx6 \| \| Epdr1 \| \| Tmem158 \| \| Ece1 \| | \| Serpinf1 \| \| --- \| \| Nrgn \| \| Mlkl \| \| Igh-VJ558 \| \| Pdzk1ip1 \| \| Runx2 \| \| Icos \| \| Chst1 \| \| Esr1 \| \| Nfic \| \| Gem \| \| Rgs3 \| \| Sesn1 \| \| Arpp21 \| \| Tpm3 \| \| Vav3 \| \| Zfp36 \| \| Carhsp1 \| \| Rab6b \| \| Gcnt2 \| \| Cd69 \| \| Gprc5b \| \| Cobll1 \| \| Fgd6 \| \| Ptpn13 \| \| Sh3tc2 \| \| Gpr125 \| \| Il16 \| \| Rasl12 \| \| Map3k14 \| \| Tnk2 \| \| Slc4a8 \| \| Paqr9 \| \| Gng2 \| \| Hoxa10 \| \| Unc5a \| \| 8430419L09Rik \| \| Mical1 \| \| Prkce \| \| Vldlr \| \| Glis2 \| \| Mboat1 \| \| Samd14 \| \| Lasp1 \| \| Atp8b2 \| \| Tmem56 \| \| Ubac1 \| \| Atp10a \| \| Tmem159 \| \| Ccne1 \| \| Mtpn \| \| \| Car7 \| \| \| Stk16 \| \| \| Rag1 \| \| \| Lhfpl2 \| \| \| Nkx2-3 \| \| \| Aim1 \| \| \| Ahnak \| \| \| Gprasp2 \| \| \| Ppat \| \| \| Sept6 \| \| | \| Syngr1 \| \| --- \| \| Ube2e2 \| \| Elk3 \| \| Cplx2 \| \| Tuba1a \| \| Zfp827 \| \| Mpl \| \| Dusp6 \| \| Slc45a3 \| \| Cxxc5 \| \| Meis1 \| \| Mllt4 \| \| Chga \| \| Slc22a18 \| \| Prdm16 \| \| Antxr2 \| \| Alpk3 \| \| Lpp \| \| Ets1 \| \| Arhgef3 \| \| B3gnt5 \| \| Rhoq \| \| Rassf2 \| \| Daam1 \| \| Eya1 \| \| Abcg1 \| \| Cmtm3 \| \| Fmnl2 \| \| 5031439G07Rik \| \| Dusp2 \| \| 1700025G04Rik \| \| Tpm4 \| \| Myct1 \| \| Acox1 \| \| Socs1 \| \| Dab2ip \| \| Emid1 \| \| Gpsm1 \| \| Camk2g \| \| Phf13 \| \| Zfp608 \| \| Nrxn1 \| \| Erg \| \| Tgfbr2 \| \| Zyx \| \| Arhgef5 \| \| 1110003E01Rik \| \| Map4k4 \| \| Coro2a \| \| Camkk1 \| \| Nav1 \| \| Smad1 \| \| Pde10a \| \| Cav2 \| \| Bysl \| \| Klf2 \| \| Kctd12 \| \| Ccdc43 \| \| Gata2 \| \| Leprel2 \| \| Ncoa3 \| \| Psmb8 \| |
