# Supplementary material for: Correlated miR-mRNA Expression Signatures of Mouse Hematopoietic Stem and Progenitor Cell Subsets Predict “Stemness” and “Myeloid” Interaction Networks
Source: PLoS One. 2014 Apr 18;9(4):e94852. doi: 10.1371/journal.pone.0094852 (PMC3991639; doi:10.1371/journal.pone.0094852)
Supplement: Table S5 — Predicted Targets of MEP miRs. 55 targets predicted by TargetScan and/or MiRanda to have binding sites for at least one “MEP miR”. Additionally, each target shows a statistically significant inverse pattern of expression with its targeting miR, across our 6 HSPC populations (p<0.05). (DOCX) [file pone.0094852.s005.docx]

**Table S5: Predicted targets of “MEP miRs”**

| **MEP Targets** | | | |
| --- | --- | --- | --- |
| \| Prdm16 \| \| --- \| \| Bach2 \| \| Trp53inp1 \| \| Mycn \| \| Meis1 \| \| Nfia \| \| Lpp \| \| Met \| \| Slc30a4 \| \| Trps1 \| \| Fmnl2 \| \| 9830001H06Rik \| \| Vldlr \| \| Snn \| | \| Arhgef3 \| \| --- \| \| Tfrc \| \| Zfhx3 \| \| Nav1 \| \| Ece1 \| \| Prkce \| \| Antxr2 \| \| Ccnk \| \| Mllt4 \| \| Cd2ap \| \| Hoxa10 \| \| Mtpn \| \| Cerk \| \| Emid1 \| | \| Glis2 \| \| --- \| \| Camk2g \| \| Gata2 \| \| Rhoq \| \| Slco3a1 \| \| Ets1 \| \| Spnb2 \| \| Nfat5 \| \| Nkx2-3 \| \| Btaf1 \| \| Zfp827 \| \| Spnb1 \| \| Stk16 \| \| Pde1b \| | \| Mllt6 \| \| --- \| \| Csf1 \| \| Rab6b \| \| Unc5a \| \| Abcg4 \| \| Carhsp1 \| \| Paqr4 \| \| Mpl \| \| 5031439G07Rik \| \| Coro2a \| \| Crim1 \| \| Psmb8 \| \| Esr1 \| |
